# Cardiac fibroblast BAG3 regulates TGFBR2 signaling and fibrosis in dilated cardiomyopathy

**DOI:** 10.1172/JCI181630

**Published:** 2025-01-02

**Authors:** Bryan Z. Wang, Margaretha A.J. Morsink, Seong Won Kim, Lori J. Luo, Xiaokan Zhang, Rajesh Kumar Soni, Roberta I. Lock, Jenny Rao, Youngbin Kim, Anran Zhang, Meraj Neyazi, Joshua M. Gorham, Yuri Kim, Kemar Brown, Daniel M. DeLaughter, Qi Zhang, Barbara McDonough, Josephine M. Watkins, Katherine M. Cunningham, Gavin Y. Oudit, Barry M. Fine, Christine E. Seidman, Jonathan G. Seidman, Gordana Vunjak-Novakovic

**Affiliations:** 1 Department of Biomedical Engineering, Columbia University, New York, New York, USA.; 2 Department of Genetics, Harvard Medical School, Boston, Massachusetts, USA.; 3 Department of Medicine, Columbia University Medical Center, New York, New York, USA.; 4 Proteomics and Macromolecular Crystallography Shared Resource, Herbert Irving Comprehensive Cancer Center, and; 5 Department of Pathology and Cell Biology, Columbia University Irving Medical Center, New York, New York, USA.; 6 Division of Cardiology, Department of Medicine, Faculty of Medicine and Dentistry, and; 7 Mazankowski Alberta Heart Institute, Faculty of Medicine and Dentistry, University of Alberta, Edmonton, Alberta, Canada.; 8 Department of Cardiology, University Heart & Vascular Center Hamburg, University Medical Center Hamburg-Eppendorf, Hamburg, Germany.; 9 German Centre for Cardiovascular Research (DZHK), Partner Site Hamburg/Kiel/Luebeck, Hamburg, Germany.; 10 Cardiovascular Division, Brigham and Women’s Hospital, Boston, Massachusetts, USA.; 11 Cardiac Unit, Massachusetts General Hospital, Boston, Massachusetts, USA.; 12 Howard Hughes Medical Institute, Bethesda, Maryland, USA.

**Keywords:** Cardiology, Cardiovascular disease, Fibrosis, Human stem cells

## Abstract

Loss of Bcl2-associated athanogene 3 (BAG3) is associated with dilated cardiomyopathy (DCM). BAG3 regulates sarcomere protein turnover in cardiomyocytes; however, the function of BAG3 in other cardiac cell types is understudied. In this study, we used an isogenic pair of BAG3-knockout and wild-type human induced pluripotent stem cells (hiPSCs) to interrogate the role of BAG3 in hiPSC-derived cardiac fibroblasts (CFs). Analysis of cell type–specific conditional knockout engineered heart tissues revealed an essential contribution of CF BAG3 to contractility and cardiac fibrosis, recapitulating the phenotype of DCM. In *BAG3^–/–^* CFs, we observed an increased sensitivity to TGF-β signaling and activation of a fibrogenic response when cultured at physiological stiffness (8 kPa). Mechanistically, we showed that loss of BAG3 increased transforming growth factor-β receptor 2 (TGFBR2) levels by directly binding TGFBR2 and mediating its ubiquitination and proteasomal degradation. To further validate these results, we performed single-nucleus RNA sequencing of cardiac tissue from DCM patients carrying pathogenic *BAG3* variants. *BAG3* pathogenic variants increased fibrotic gene expression in CFs. Together, these results extend our understanding of the roles of BAG3 in heart disease beyond the cardiomyocyte-centric view and highlight the ability of tissue-engineered hiPSC models to elucidate cell type–specific aspects of cardiac disease.

## Abstract 2

Using a combination of tissue engineering, molecular biology, and next generation sequencing approaches, we gain insight into the function of BAG3 in human cardiac fibroblasts.

## Graphical abstract

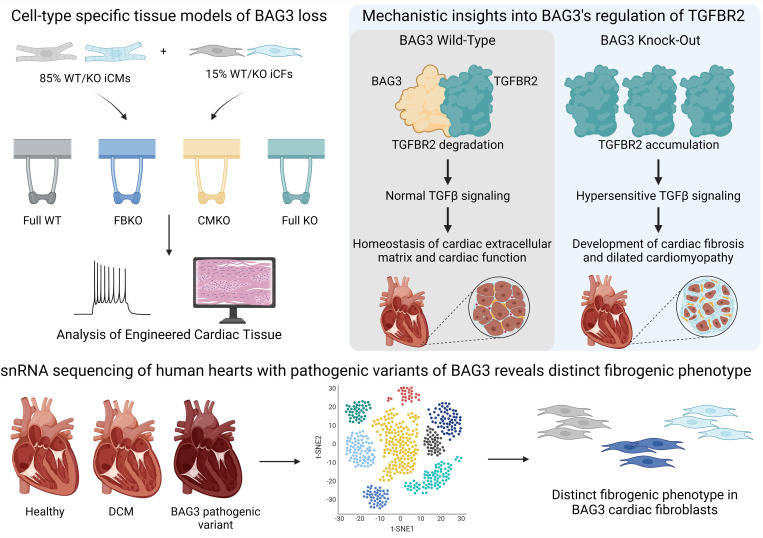

## Introduction

Bcl2-associated athanogene 3 (BAG3) is a co-chaperone protein highly expressed in heart and skeletal muscle. Initial studies demonstrated early lethality in homozygous *Bag3-*knockout mice (1). Since then, BAG3 has emerged as a critical regulator of cardiac proteostasis, and BAG3 mutations have been identified as a cause of genetic dilated cardiomyopathy (DCM) (2–4).

In the sarcomere, BAG3 forms a ternary complex with heat shock protein 70 (HSP70) and small heat shock protein B8 (HSPB8) and aids in the removal of proteins denatured by mechanical stress (5–8). Despite these mechanistic insights, our understanding of BAG3 in the heart is limited to its function in cardiomyocytes (CMs), while BAG3 is a ubiquitously expressed protein. Recent single-nucleus sequencing data show that CMs constitute a minority (30%–50%) of the heart by number, emphasizing the importance of the contribution of non-myocytes in homeostasis (9–11). Of particular interest are cardiac fibroblasts (CFs), which are the primary mediators of extracellular matrix (ECM) composition (12, 13). Fibroblasts remodel the heart in disease, leading to ventricle wall thinning, chamber dilation, and loss of ventricle stiffness (14). Traditionally, these changes in the ECM are viewed as a compensatory process in response to CM death. However, given its high expression in CFs, we posit there may be an intrinsic, deleterious effect of BAG3 loss in CFs.

The study of CF biology is limited by the lack of specific markers for the isolation of CFs from explanted tissue, which hinders assessment of pure populations of CFs at the cellular level (15). To address this, several robust protocols for CF differentiation from human induced pluripotent stem cells (hiPSCs) have been reported (16–18). In addition, advances in cardiac tissue engineering allow for noninvasive, imaging-based measurements of contractile force and promote the maturation of hiPSC-CMs in a 3D microenvironment (19, 20). Our laboratory and others have demonstrated the ability of hiPSC-based engineered tissues to model cardiac development, elucidate normal and pathological function, and parse out the respective contributions of CMs and supporting cells (21–25).

Here, we used isogenic hiPSC-derived fibroblasts to study the role of BAG3 in CFs. By generating cell type–specific conditional knockout (cKO) engineered cardiac tissues, we showed that the loss of CF BAG3 is detrimental to tissue function and recapitulates the phenotypic hallmarks of DCM. Mechanistically, we show that BAG3 is a negative regulator of type II TGF-β receptor (TGFBR2) via proteasomal degradation. Finally, by conducting single-nucleus RNA sequencing (snRNA-Seq) of cardiac tissue from BAG3 cardiomyopathy patients, we observed that pathogenic variants in *BAG3* driving DCM activate a fibrogenic program in cardiac fibroblasts.

## Results

### Cell type–specific contribution of BAG3 to engineered cardiac tissue function.

In cardiac tissue lysates from human DCM patients, we observed a global decrease of BAG3 compared with controls of non-failing hearts, consistent with previous studies (Supplemental Figure 1A; supplemental material available online with this article; https://doi.org/10.1172/JCI181630DS1) (7). To isolate the specific role of BAG3 in cardiac fibroblasts (CFs), we studied *BAG^+/–^* and *BAG3^–/–^* hiPSCs, which were provided by Bruce Conklin (Gladstone Institutes, UCSF, San Francisco, California, USA) (26, 27). We differentiated the *BAG3^–/–^* hiPSCs and their wild-type (WT; *BAG3^+/+^*) isogenic control (the WTC11 cell line) into CFs using a previously established method (Supplemental Figure 1, B and C) (16). During differentiation, CFs undergo an epicardial cell progenitor state marked by zona occludens 1 (ZO1) and WT1 transcription factor (WT-1) staining and, once specified, express vimentin and connexin 43 (Supplemental Figure 1D). We confirmed the CF-specific markers transcription factor 21 (TCF21) and discoidin domain receptor tyrosine kinase 2 (DDR2) by immunofluorescence in *BAG3^+/+^* and *BAG3^–/–^* CFs (Supplemental Figure 1E). CFs had similar BAG3 expression compared with differentiated CMs from the same hiPSC lines, and BAG3 had cytoplasmic distribution within the CFs (Supplemental Figure 1, F and G).

**Figure 1:**
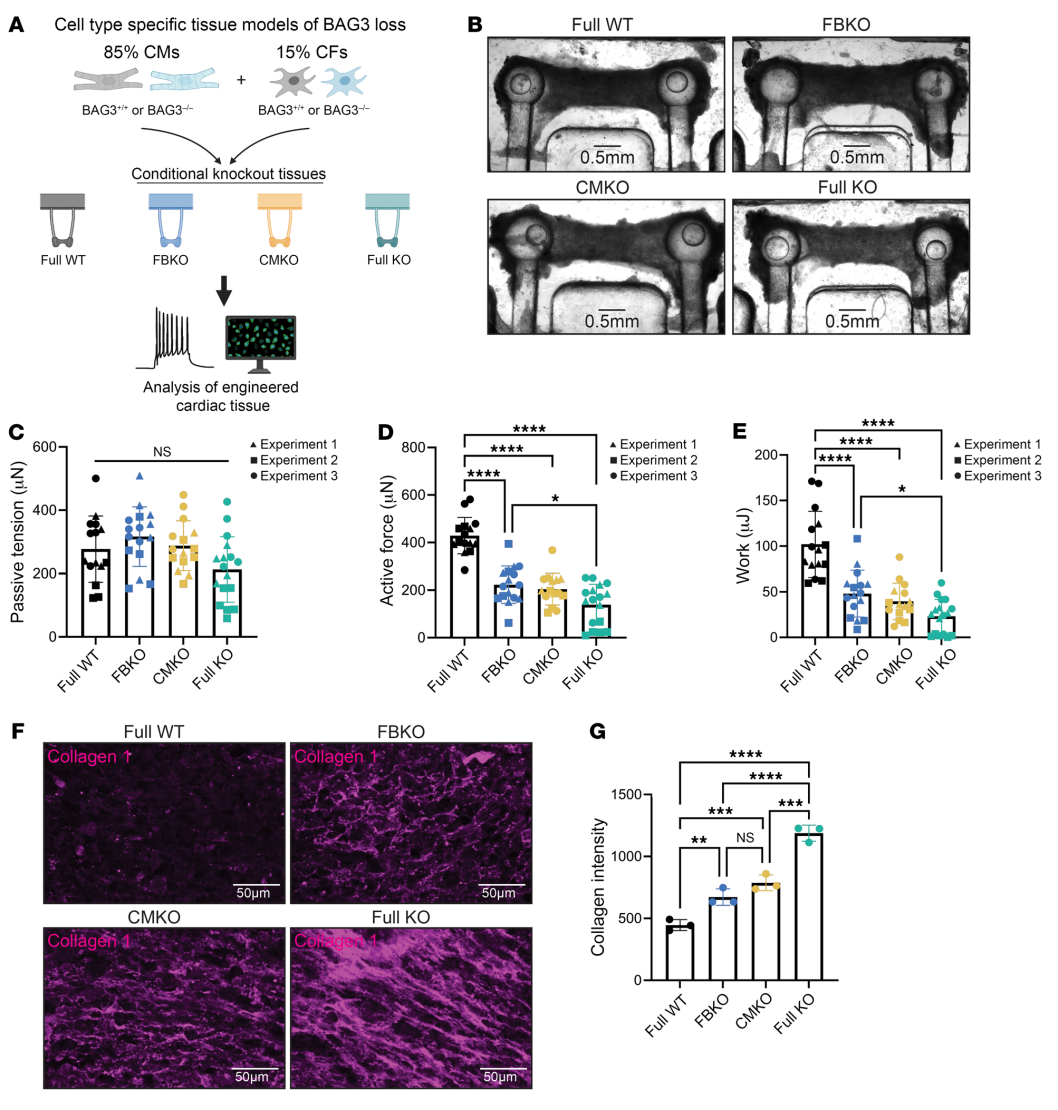
Engineered cardiac tissues reveal the contribution of cardiac fibroblast BAG3 to cardiac function. (**A**) Schematic of experimental design. (**B**) Representative bright-field microscopy of engineered cardiac tissues. Scale bars: 0.5 mm. (**C**–**E**) Measurements of cardiac tissue passive tension (**C**), contractile force (**D**), and work (**E**). (**F** and **G**) Immunofluorescence staining of cardiac tissues with collagen I (**F**) and quantification (**G**). Scale bars: 50 μm. *P < 0.05, **P < 0.01, ***P < 0.001, ****P < 0.0001 by 1-way ANOVA with post hoc Tukey’s test. Functional data pooled from 3 independent experiments, resulting in *n* = 15–17 per genotype.

To model the cell type–specific contribution of BAG3 to cardiac function, we leveraged our engineered cardiac tissue platform and created a series of isogenic cKO tissues: (a) wild-type (CM^+/+^, CF^+/+^; Full WT), (b) fibroblast knockout (CM^+/+^, CF^–/–^; FBKO), (c) cardiomyocyte knockout (CM^–/–^, CF^+/+^; CMKO), and (d) full knockout (CM^–/–^, CF^–/–^; Full KO) (Figure 1A). hiPSC-CMs (>95% TNNT2^+^ by flow cytometry; Supplemental Figure 1H) were combined with hiPSC-CFs in an 85%/15% ratio and encapsulated in a fibrin hydrogel cross-linked with thrombin. We selected this initial percentage of fibroblasts as it has been reported in both human datasets and lineage tracing experiments in mice (28–31). After tissue formation, tissues were matured by ramped electrical stimulation based on our previous studies. BAG3 loss did not affect the cells’ ability to form compact tissues (Figure 1B). Tissue passive (diastolic) tension did not significantly differ, indicating no hypercontractile phenotype observed in other tissue models of hypertrophic or restrictive cardiomyopathy (Figure 1C) (32).

All cKO tissues demonstrated decreased active contractile force generation (Figure 1D). To normalize for the variability in passive tension and pillar starting position across tissues, we calculated the work performed by each tissue, which was decreased in all cKO tissues (Figure 1E), with the lowest work produced by the Full KO group. Given the known role of BAG3 in sarcomere integrity, the reduction in force in CMKO and Full KO tissues was unsurprising. However, the loss of BAG3 in CFs impaired normal CM function in the FBKO group, and we asked whether this was due to an increase in ECM deposition in the cardiac tissues. Indeed, we observed increased collagen deposition that inversely correlated with force data (Figure 1, F and G). CMKO tissues exhibited increased fibrosis, in line with previous work that established fibrosis as a sequela of the deletion of CM *Bag3* (33). FBKO also led to greater cytotoxicity compared with Full WT, measured by lactate dehydrogenase (LDH) extravasation into culture medium (Supplemental Figure 1I).

While recent reports differ on whether or not fibroblast cell proportion expands in disease (28, 34), we formally tested the effect of FBKO at higher fibroblast percentages in our tissues by increasing the ratio during tissue formation to 40% and 60% fibroblasts. We found that increasing the fibroblast percentage to 40% increased the force generation of WT tissues, in line with previous studies demonstrating the critical role of non-myocyte cells in engineered cardiac tissue contractility (35–37). Critically, we showed reduced force production and increased collagen deposition in the FBKO group compared with full WT in tissues of every ratio (Supplemental Figure 1J-L). Taken together, these data suggest a role of CF BAG3 in maintaining cardiac contractility and ECM composition.

### Loss of BAG3 alters the CF proteome.

**Figure 2:**
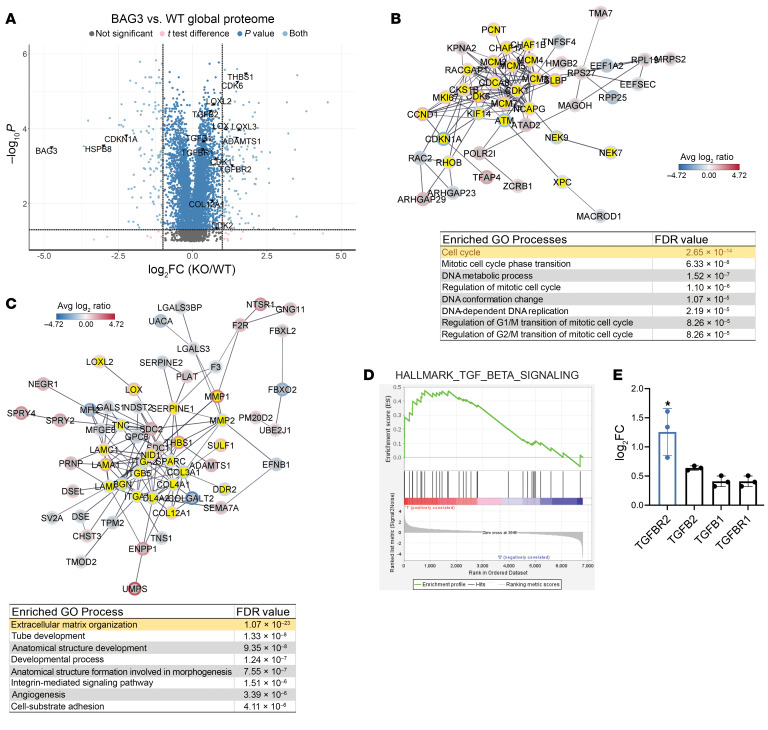
Loss of BAG3 alters CF phenotype. (**A**) Differentially expressed proteins in *BAG3^–/–^* CFs. (**B** and **C**) STRING analysis of protein-protein interactions reveals 2 clusters of cell cycle– and ECM-associated protein networks. (**D**) Gene set enrichment analysis of TGF-β signaling pathway. (**E**) Fold change values for the TGF-β ligands and receptors identified by mass spectrometry. *P < 0.05 by 2-tailed Student’s *t* test with permutation-based FDR correction less than 0.05. *n* = 3 independent differentiations in mass spectrometry data.

We next analyzed *BAG3^+/+^* and *BAG3^–/–^* CFs’ global proteome by mass spectrometry in isolated 2-dimensional (2D) culture. *BAG3^–/–^* CFs had significantly different expression of 766 proteins compared with *BAG3^+/+^* (cutoffs: FDR = 0.05 and log_2_ fold change [log_2_FC] = 0.58) (Figure 2A and Supplemental Table 1).

Network analysis of protein-protein interactions of significant proteins using the STRING database identified 2 major clusters enriched in cell cycle– and ECM-related processes (Figure 2, B and C). Cell cycle proteins such as p21 (CDKN1A) and cyclin-dependent kinases (CDK1/2/6) were differentially expressed. Indeed, proliferation rate was increased in *BAG3^–/–^* CFs compared with *BAG3^+/+^* CFs as quantified by EdU staining (Supplemental Figure 2A). To exclude the possibility that clonal selection of hiPSCs contributed to this phenotype, we further assayed heterozygous *BAG^+/–^* CFs. Ki-67 staining revealed a dose-dependent effect of loss of BAG3, suggesting that the increase in fibroblast proliferation is a biological effect of BAG3 loss (Supplemental Figure 2B). These results align with previous studies suggesting BAG3’s role in the cell cycle and mitosis (38, 39).

In the ECM-related cluster, levels of thrombospondin 1 (THBS1) and lysl oxidases responsible for collagen cross-linking (LOX, LOXL2, LOXL3) were significantly increased in *BAG3^–/–^* CF. Gene set enrichment analysis further revealed an increase in the TGF-β signaling pathway in *BAG3^–/–^* CFs (Figure 2D) (40). *BAG3^–/–^* CFs expressed significantly more type II TGF-β receptor (TGFBR2; log_2_FC = 1.25) (Figure 2E), which was confirmed by Western blotting (Supplemental Figure 2, C and D). Interestingly, TGF-β ligand was not significantly upregulated. Assaying cell culture supernatant using ELISA, we observed an increase in TGF-β1 secretion by knockout CMs, but not knockout CFs (Supplemental Figure 2E). This suggested augmentation of ligand and receptor interactions between CMs and CFs and may explain the fibrosis observed in our CMKO model. Together, these data indicate that the loss of BAG3 significantly alters CF phenotype and the TGF-β pathway, particularly at the level of the receptor.

### BAG3 loss sensitizes the TGF-β signaling cascade response through TGFBR2.

**Figure 3:**
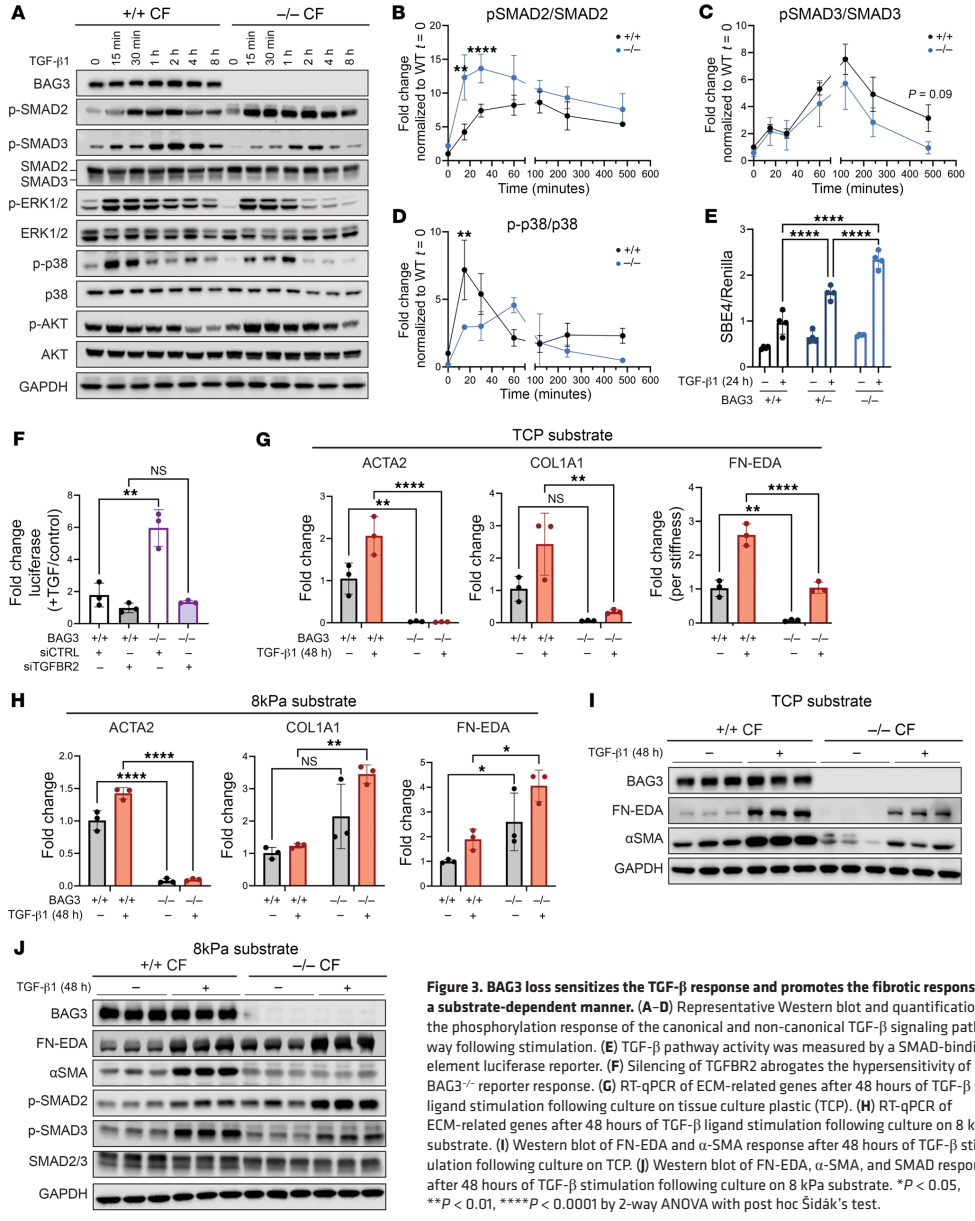
BAG3 loss sensitizes the TGF-β response and promotes the fibrotic response in a substrate-dependent manner. (**A**–**D**) Representative Western blot and quantification of the phosphorylation response of the canonical and non-canonical TGF-β signaling pathway following stimulation. (**E**) TGF-β pathway activity was measured by a SMAD-binding element luciferase reporter. (**F**) Silencing of TGFBR2 abrogates the hypersensitivity of BAG3^–/–^ reporter response. (**G**) RT-qPCR of ECM-related genes after 48 hours of TGF-β ligand stimulation following culture on tissue culture plastic (TCP). (**H**) RT-qPCR of ECM-related genes after 48 hours of TGF-β ligand stimulation following culture on 8 kPa substrate. (**I**) Western blot of FN-EDA and α-SMA response after 48 hours of TGF-β stimulation following culture on TCP. (**J**) Western blot of FN-EDA, α-SMA, and SMAD response after 48 hours of TGF-β stimulation following culture on 8 kPa substrate. *P < 0.05, **P < 0.01, ****P < 0.0001 by 2-way ANOVA with post hoc Šidák’s test.

TGFBR2 forms heterodimeric complexes with coreceptors that bind ligand and transduce signaling through canonical and non-canonical pathways (41–43). We thus aimed to characterize the consequences of increased TGFBR2 expression in CFs. Cell membrane biotinylation analyses of CFs demonstrated increased TGFBR2 at the cell membrane, accessible to bind ligand (Supplemental Figure 2F). We then tested the sensitivity of these cells to TGF-β ligand stimulation. Treatment with TGF-β1 ligand over 8 hours revealed significant differences in the phosphorylation events downstream of TGFBR2 in *BAG3^–/–^* CFs compared with *BAG3^+/+^* (Figure 3A). In the canonical pathway, SMAD2 phosphorylation was significantly increased, both at baseline and following stimulation (Figure 3B). On the other hand, SMAD3 phosphorylation showed a downward trend (Figure 3C). In the non-canonical pathway, we observed a loss of maximal p38 phosphorylation in *BAG3^–/–^* CFs (Figure 3D).

Assessment of the canonical TGF-β pathway transcriptional activity using a SMAD-binding element (SBE4) luciferase reporter revealed a dose-dependent increase in TGF-β sensitivity between *BAG^+/+^*, *BAG^+/–^*, and *BAG^–/–^* CFs (Figure 3E). Cotransfection with TGFBR2-specific siRNA negated the increased luciferase transcription in response to TGF-β ligand (Figure 3F). Notably, siTGFBR2 also attenuated the proliferative phenotype in *BAG3^–/–^* CFs (Supplemental Figure 2G). Therefore, the measured increase in TGFBR2 also corresponded with increased pathway activity.

### Decoupling mechanical and ligand-mediated fibroblast activation reveals a fibrogenic program in BAG3^–/–^ CFs.

We next tested ECM remodeling in response to TGF-β ligand. Surprisingly, assessment of the myofibroblast activation marker α-smooth muscle actin (ACTA2/α-SMA), collagen I (COL1A1), and the disease-associated fibronectin EDA isoform (FN-EDA) by quantitative PCR showed blunted expression in *BAG^–/–^* CFs (Figure 3G) (44, 45). This was puzzling, as it suggested an antifibrotic effect of BAG3 loss, which conflicted with the results of our engineered tissue model and known disease phenotype.

A critical difference between engineered cardiac tissues and traditional culture is the cells’ surrounding substrate. Tissue culture plastic is known to induce the activation of fibroblasts owing to its supraphysiological stiffness (10^7^ kPa), compared with the 1–20 kPa of the native heart (46). BAG3 has established functions as a mechanosensor, and we hypothesized that the phenotype we observed was due to changes in such signaling (47, 48). Therefore, to separate the factors of mechanical activation from ligand activation, we cultured the cells atop soft polydimethylsiloxane at physiological stiffness (8 kPa). The soft substrate normalized the gene expression of FN-EDA and COL1A1 between genotypes, and we observed that these markers were more sensitive to TGF-β stimulation in *BAG3^–/–^* CFs (Figure 3H). We validated these findings at the protein level using Western blot and quantification of total collagen in the cell culture supernatant, further demonstrating the ECM-secreting state of *BAG3^–/–^* CFs (Figure 3, I and J, and Supplemental Figure 3, A and B). *BAG3^–/–^* CFs again displayed decreased ACTA2/α-SMA gene expression and protein expression, suggesting that this phenotype is independent of the classic myofibroblast cell state. Nevertheless, the hyperphosphorylation of SMAD2 but not SMAD3 persisted (Figure 3J and Supplemental Figure 3, C and D), and silencing of TGFBR2 reduced FN-EDA mRNA levels in *BAG3^–/–^* CFs grown on 8 kPa substrates (Supplemental Figure 3E). These data suggest that TGFBR2 upregulation after BAG3 loss induces a fibrogenic cell state at physiological stiffness, consistent with our engineered cardiac tissue model.

### BAG3 regulates the proteasomal degradation of TGFBR2.

**Figure 4:**
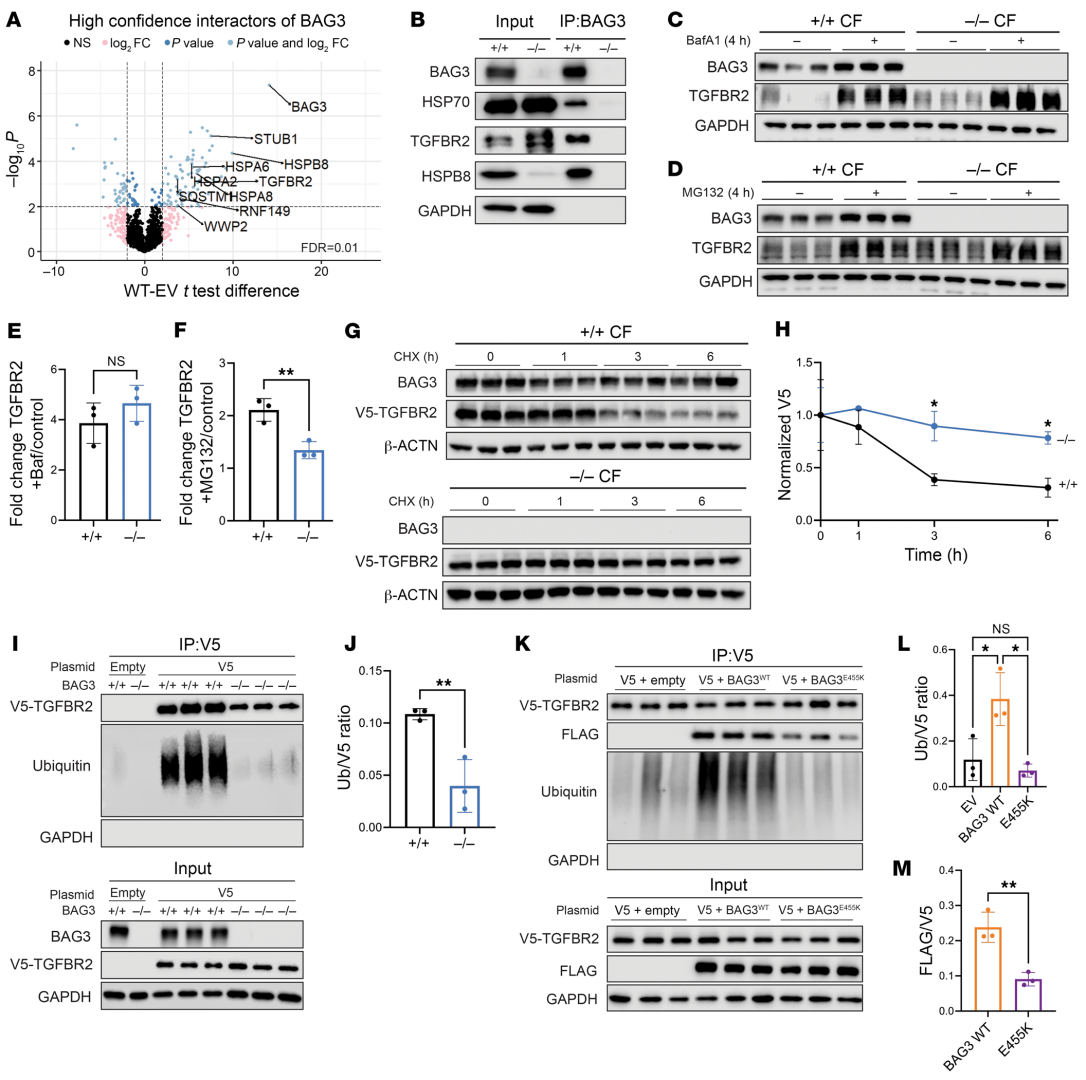
BAG3 binds TGFBR2 and mediates its proteasomal degradation. (**A**) Identification of BAG3 binding partners in CFs using affinity purification of FLAG and mass spectrometry (AP-MS). (**B**) Immunoprecipitation of endogenous BAG3 confirms TGFBR2 interaction. (**C**) Lysosomal flux of TGFBR2 measured by Western blot. (**D**) Proteasomal flux of TGFBR2 measured by Western blot. (**E**) Quantification of **C**. (**F**) Quantification of **D**. (**G** and **H**) Cycloheximide (CHX) chase of V5-tagged TGFBR2 (**G**) and quantification (**H**). (**I** and **J**) Ubiquitination assay of V5-TGFBR2 (**I**) and quantification (**J**). (**K** and **L**) Rescue of TGFBR2 ubiquitination in BAG3^–/–^ background (**K**) and quantification (**L**). (**M**) BAG3^E455K^ reduces TGFBR2 and BAG3 binding. *P < 0.05, **P < 0.01 by unpaired 2-tailed Student’s *t* test (**E**, **F**, and **J**), 1-way ANOVA with post hoc Tukey’s test (**L** and **M**), or 2-way ANOVA with post hoc Šidák’s test (**H**). *n* = 3 independent transfections.

BAG3 is a scaffolding protein that links binding partners with a variety of molecular chaperones and ubiquitin ligases to mediate protein degradation (49). Thus, we hypothesized that BAG3 could modulate TGFBR2 through a similar mechanism. We overexpressed a 3xFLAG-tagged BAG3 or an empty vector in *BAG3^–/–^* CFs to rescue proteins from altered proteostasis. Affinity purification of FLAG and mass spectrometry (AP-MS) identified 59 high-confidence interactors of BAG3, with a false discovery rate (FDR) of 0.01 (Figure 4A and Supplemental Table 2). Consistent with previous reports, BAG3 interacted with the heat shock protein 70 (HSP70) family (HSPA1, HSPA2, HSPA6, HSPA8), HSPB8, and the autophagy adaptor SQSTM1/p62, as well as the E3 ligase C-terminus of HSC70-interacting protein (CHIP/STUB1) (7, 50).

Notably, we identified TGFBR2 as an interacting partner of BAG3 in CFs, which we then confirmed by coimmunoprecipitation analysis of endogenous BAG3 (Figure 4B). This was an intriguing finding since TGFBR2 levels are tightly regulated through proteasome and lysosomal pathways (endocytic and autophagic, respectively) (51). By analogy to previous studies in CMs, which showed a role for BAG3 in autophagy, we suspected a deficit in the autophagic turnover of TGFBR2 (7, 52). Indeed, inhibition of lysosomal acidification using bafilomycin A1 (BafA1) showed a decrease of total autophagic flux in *BAG3^–/–^* CFs, identified by the levels of lipidated LC3B (Supplemental Figure 3F). However, the flux of TGFBR2 through lysosomal pathways was not different between *BAG3^+/+^* and *BAG3^–/–^* CFs (Figure 4, C and E). Conversely, inhibition of the proteasome with MG-132 revealed a significant decrease in the flux of TGFBR2 in *BAG3^–/–^* CFs compared with *BAG3^+/+^* (Figure 4, D and F). Pulse-chase experiments with the translation inhibitor cycloheximide showed a significantly longer decay time of transfected V5-tagged TGFBR2 in *BAG3^–/–^* CFs (Figure 4, G and H). These data suggest that BAG3 regulates the expression of TGFBR2 through its degradation at the proteasome.

These findings could be due to either a global deficit in proteasome activity or a specific lack of TGFBR2 turnover in *BAG3^–/–^* CFs. To test this, we assessed global proteasome proteolytic activity by measuring the cleavage of fluorescent substrate in cell lysate. Paradoxically, *BAG3^–/–^* CFs showed increased proteasome activity compared with *BAG3^+/+^* (Supplemental Figure 3G).

Next, we tested the proteasomal targeting of TGFBR2 by evaluating its ubiquitination. Since V5-TGFBR2 was more stable in *BAG3^–/–^* CFs, we titrated plasmid overexpression to obtain equivalent pull-down between genotypes (Supplemental Figure 3H). *BAG3^–/–^* CFs had significantly lower ubiquitination of immunoprecipitated V5-TGFBR2 compared with WT (Figure 4, I and J). There was no difference in global ubiquitin between the cell lines, suggesting a specific difference in TGFBR2 (Supplemental Figure 3I). We further sought to rescue ubiquitination via cotransfection of BAG3 in *BAG3^–/–^* CFs. BAG3^WT^, but not the DCM-causing mutant *BAG3^E455K^*, rescued the ubiquitination of V5-TGFBR2 (Figure 4, K and L). Additionally, the interaction between TGFBR2 and BAG3^E455K^ was diminished (Figure 4M). Because the E455K mutation disrupts binding of BAG3 with HSP70 (Supplemental Figure 3J), it is likely that the BAG3-HSP70 interaction is required for TGFBR2 ubiquitination as well as to stabilize the BAG3-TGFBR2 complex.

Finally, we assessed the role of BAG3 in primary human cardiac fibroblasts via BAG3 knockdown (Supplemental Figure 4A). Silencing of BAG3 increased COL1A1 and FN-EDA mRNA expression both at baseline and after treatment with TGF-β ligand for 48 hours on 8 kPa substrate (Supplemental Figure 4, B and C). Moreover, BAG3 knockdown increased collagen I secretion and FN-EDA expression at the protein level (Supplemental Figure 4, D–H). Mirroring our findings in iPSC-CFs, siBAG3-treated cells had hypersensitive SMAD2 phosphorylation after TGF-β ligand stimulation for 15 minutes (Supplemental Figure 4, I and J). Together, these data convincingly establish BAG3 as a regulator of TGFBR2 through protein degradation in the cardiac fibroblast.

### Human mutations in BAG3 and BAG3^–/–^ CFs converge on TGF-β signaling.

**Figure 5:**
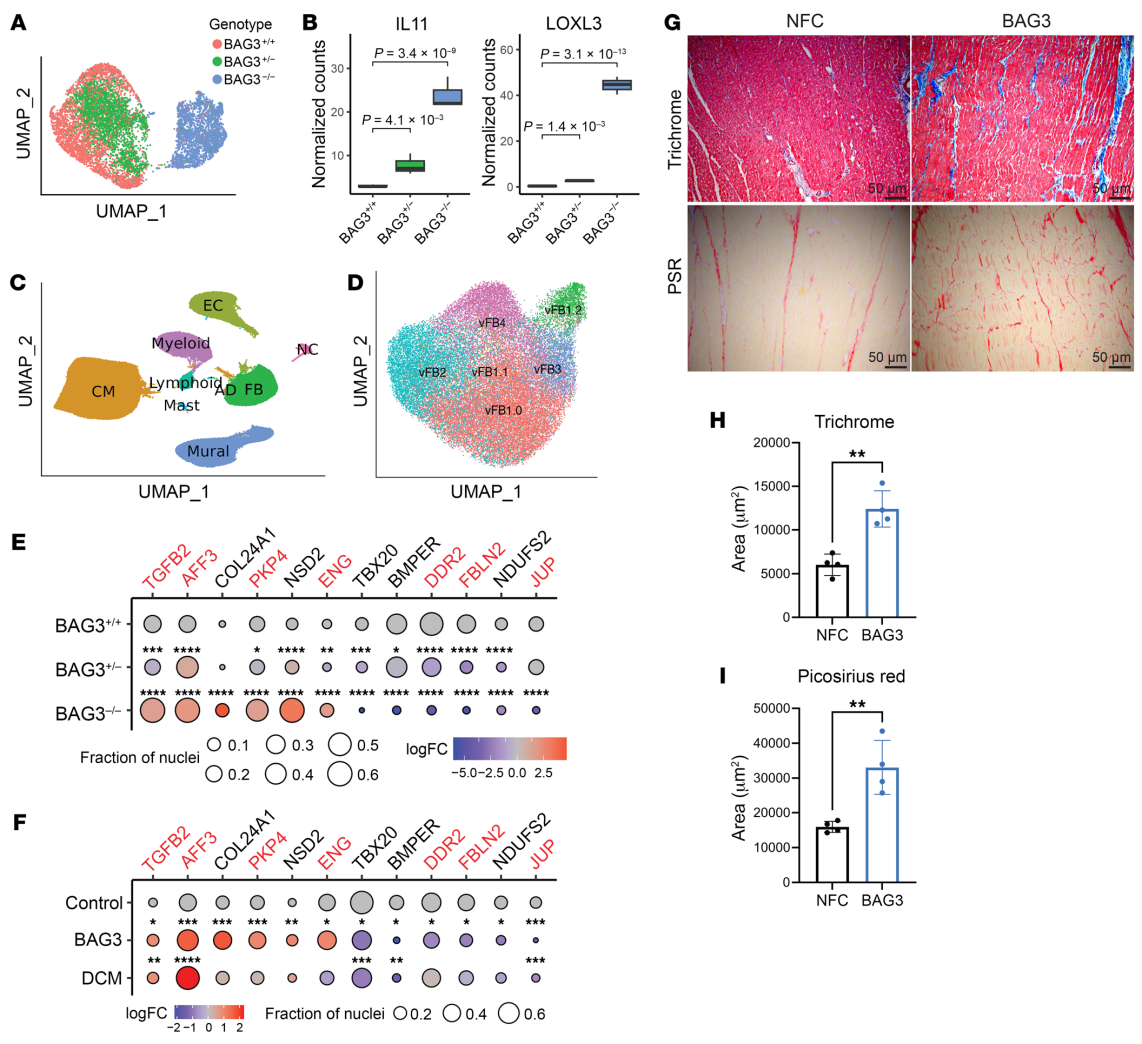
Gene expression changes in *BAG3* fibroblasts indicate increased TGF-β signaling. (**A**) Uniform manifold approximation and projection (UMAP) visualization of multiplexed snRNA-Seq data of cultured CFs, color-coded based on their respective genotypes. (**B**) Averaged and normalized expression of fibrosis- and TGF-β–related genes. Median values are denoted by black horizontal bars. The interquartile range is illustrated by the upper and lower boundaries of the box. The highest and lowest values are indicated by the top and bottom ends of the vertical lines. Adjusted *P* values from edgeR analysis are provided at the top. (**C**) UMAP of non-failing control and DCM-affected left ventricles, distinguished by their assigned cell types. (**D**) UMAP of fibroblasts, with color indicating their respective cell states. (**E** and **F**) Dot plot illustrating the differentially expressed genes in *BAG3^+/+^*, *BAG3^+/–^*, and *BAG3^–/–^* hiPSC-CFs (**E**) and human tissue fibroblasts (**F**). Genes highlighted in red indicate significantly differential expression in the proteomics of *BAG3^–/–^* hiPSC-CFs. The size of each dot represents the fraction of nuclei expressing each gene, and colors indicate log_2_ fold change. Adjusted *P* values from edgeR analysis are displayed on top of each dot. *P < 0.05, **P < 0.01, ***P < 0.001, ****P < 0.0001. (**G**) Representative images from staining of explanted BAG3 DCM (*n* = 4) hearts and non-failing control (NFC) hearts (*n* = 4) with Masson’s trichrome and Picrosirius red (PSR) staining. Scale bars: 50 μm. (**H**) Quantification of fibrosis in trichrome images. Each dot represents the average fibrosis present in patient samples from several histological slices. *n* = 4 for both NFC and BAG3-mutant DCM patients. (**I**) Quantification of fibrosis in PSR images. *n* = 4 for both NFC and BAG3-mutant DCM patients. **P < 0.01 by 2-tailed Student’s *t* test (**H** and **I**).

To further validate our model, we conducted multiplexed snRNA-Seq (53) on 3 independent differentiations of *BAG3^+/+^*, *BAG3^+/–^*, and *BAG3^–/–^* CFs cultured on the 8 kPa substrate (Figure 5A and Supplemental Figure 5A). Differentially expressed genes (DEGs) in *BAG3^+/–^* (*n* = 973) and *BAG3^–/–^* CFs (*n* = 5,229) exhibited significant overlap (*n* = 663, *P* = 5.08 × 10^–15^, hypergeometric test), as did the differentially expressed proteins (*n* = 289, *P* = 0.024 for *BAG3^–/–^* snRNA-Seq vs. mass spectrometry; *n* = 67, *P* = 6.53 × 10^–8^ for *BAG^+/–^* snRNA-Seq vs. mass spectrometry; hypergeometric test), indicating shared transcriptomic changes between the two cell lines (Supplemental Figure 5B). DEGs in *BAG3^+/–^* and *BAG3^–/–^* CFs that overlapped with differentially expressed proteins in *BAG3^–/–^* CFs (*n* = 59) included cell cycle–related genes (cyclin-dependent kinase 1, marker of proliferation Ki-67) and ECM component genes (fibulin 2, EGF-containing fibulin-like extracellular matrix protein 1, Fraser extracellular matrix complex subunit 1) (Supplemental Figure 5C). Notably, *TGFBR2* mRNA expression remained unchanged in *BAG3^–/–^* CFs, suggesting that post-transcriptional regulation drives increased TGFBR2 expression (Supplemental Figure 5C). Interleukin-11 (IL-11), a profibrotic ligand induced by TGF-β stimulation in CFs, and lysyl oxidase–like 3, another gene downstream of TGF-β signaling, were upregulated in a dose-dependent manner in *BAG3^+/–^* and *BAG3^–/–^* CFs (Figure 5B) (54, 55). Overall, transcriptomic changes in *BAG3^+/–^* and *BAG3^–/–^* CFs converge on increased proliferation and profibrotic gene expression.

We next sequenced tissue from the left ventricles (LVs) of 4 DCM patients harboring pathogenic variants in *BAG3* and analyzed them in the context of previously published snRNA-Seq data (28). Three patients carried premature truncating variants, and one patient carried a 4.2 kb deletion encompassing the promoter and first exon of *BAG3* (Supplemental Table 3) (56). These data were compared with region-matched snRNA-Seq data from non-failing control hearts (*n* = 14) and other DCM cohorts (comprising patients with *TTN* and *LMNA* variants, collectively labeled as DCM below; *n* = 18) (Figure 5C) (28). The identified cell types, along with their respective states and the distribution of nuclei across these states in the *BAG3* LVs, mirrored those observed in other DCM-affected LVs (Figure 5D and Supplemental Figure 6, A–C and E). *BAG3* expression was detected at low levels across all cell types (Supplemental Figure 6D).

We then analyzed gene expression changes in all fibroblasts and within each state (Supplemental Figure 6F). No significant overlap was observed between the DEGs in *BAG3^+/–^* or *BAG3^–/–^* hiPSC-CFs and the DEGs in *BAG3* fibroblasts (overlapping genes: *n* = 43 for *BAG3^+/–^*; *n* = 265 for *BAG3^–/–^*; Supplemental Figure 6H). However, consistent alterations in the expression of profibrotic genes (tissue inhibitor of metalloproteinases 3, TGF-β2, platelet-derived growth factor subunits A and D) were evident in all fibroblasts affected by DCM, including those in *BAG3* hearts (Supplemental Figure 6, G and I) (28, 57, 58). In addition, we noted shared transcriptomic changes between *BAG3* hiPSC-CFs and human fibroblasts (Figure 5, E and F, and Supplemental Figure 6I), several of which were *BAG3* specific. In particular, endoglin (*ENG*), a receptor for TGF-β ligand and a downstream target of angiotensin II (59, 60), was significantly upregulated in all *BAG3* fibroblasts but not their DCM and WT controls. Conversely, discoidin domain receptor tyrosine kinase 2 (*DDR2*) and fibulin 2 (*FBLN2)*, common marker genes for fibroblasts, were paradoxically downregulated in *BAG3* fibroblasts and hiPSC-CFs (55, 61–65). Lastly, we stained *BAG3* LVs and non-failing control LVs with Masson’s trichrome and Picrosirius red and quantified fibrosis. We observed increased fibrosis and collagen deposition in *BAG3* LVs (Figure 5, G–I). The cumulative evidence from these gene and protein expression alterations, in conjunction with the increased *TGFB2* expression, implies heightened TGF-β signaling and, importantly, concordance between primary human heart fibroblasts and hiPSC-CFs carrying *BAG3* mutations.

## Discussion

Proteostasis in the mechanically active environment of the heart is disrupted in cardiac pathology (66–68). The role of BAG3 is highlighted by its link to DCM. Here, we present evidence that the loss of BAG3 in CFs alters TGF-β signaling and drives cardiac fibrosis.

By generating cKO tissues and measuring force generation in situ, we observed that all cKO tissue models recapitulated hallmarks of clinical DCM, including a decrease in contractile function and tissue fibrosis. Importantly, tissues composed with *BAG3^–/–^* CFs showed decreased tissue contractility and increased tissue fibrosis, regardless of CM genotype. Our model captured the specific contributions of CM and CF BAG3 to tissue fibrosis, matching previously published in vivo data (33). Interestingly, the loss of BAG3 in the FBKO group caused a significant decrement in function, equal to that in the CMKO group. Considering the similar amounts of collagen deposition between FBKO and CMKO tissues, fibrosis may account for much of this effect. Indeed, we observed complementary increases in TGF-β ligand and TGFBR2 in CMs and fibroblasts, respectively. This synergy likely explains the highest level of collagen deposition in the Full KO group, concomitant with the lowest work production.

The effect of FBKO across tissues of several different compositions was robust, and the loss of BAG3 in CFs in just 15% of the tissue can inhibit the force production of WT CMs. However, it is interesting that we did not observe a dose-dependent effect on contractility when increasing fibroblast percentage. While this may be due to the limitations of our experimental model and the sensitive range of force measurement, these data may also suggest that there are ECM-independent mechanisms that contribute to the decreased contractile function in the FBKO group. It is well established that fibroblast-CM communication through paracrine signaling and electrical coupling influences CM function (69, 70). While these interactions are outside the scope of this study, there is evidence of an inflammatory milieu marked by increased IL-11 RNA expression and increased lactate dehydrogenase release in the FBKO group. Altogether, these data demonstrate the power of engineered models to capture physiologically relevant effects in vitro at much shorter time scales when compared with in vivo models.

Switching to 2D culture, we uncovered a significant difference between the ECM-secreting phenotypes in 2D that was dependent on substrate stiffness. On tissue culture plastic, the loss of BAG3 appears to dampen ECM secretion in response to TGF-β ligand stimulation; however, using soft substrate to decouple mechanical activation, we observed an inversion of this phenotype. We did not establish a mechanism for how this switch occurs in our model. Yet our findings are in line with several studies of the context-dependent functions of TGF-β signaling. For example, in the early stages of tumorigenesis, TGF-β first acts as a tumor suppressor, inhibiting proliferation (71). In later stages, it enhances survival and cell invasion (72). Notably, a previous study demonstrated that matrix stiffness regulated a switch between these disparate TGF-β functions through downregulation of the PI3K/Akt pathway (73). In addition to these possibilities, BAG3’s previously described role in mediating mechanotransduction through Hippo/YAP signaling may also play a role in response to matrix stiffness. Our data challenge several studies that have suggested the antifibrotic action of BAG3 loss, all of them using traditional culture substrates (74–76).

Mechanistically, we show that loss of BAG3 drives upregulation of TGFBR2 in a ubiquitin-dependent manner. Furthermore, the HSP70-BAG3 interaction is integral to this process, evidenced by rescue studies using BAG3^E455K^ overexpression. Interestingly, hypersensitive TGFBR2 activation and ECM secretion occurred in the notable absence of concomitant ACTA2/α-SMA expression. In our model, we observed a decrease in both p38 and SMAD3 signaling. Previous work suggests that both are required for the activation of ACTA2^+^ myofibroblasts, which may explain the differences we encountered (13, 77). The myofibroblast state is well known to secrete ECM; however, lineage tracing experiments suggest that ACTA2/α-SMA only labels a subset (15%–25%) of fibrosis-related CFs in mice (78, 79). Moreover, the function of α-SMA is primarily to generate cell tension, rather than to secrete ECM (80). While these processes often intertwine, they appear to be independent in our model.

To extend the validity of our findings in vitro, we generated an snRNA-Seq dataset from heart failure patients harboring *BAG3* mutations. Similarly to previous key works that studied the cell composition of hearts in health and disease, we observed significant alterations in fibroblast transcriptome along with evidence of TGF-β activation (9, 11, 28, 34, 81, 82). Biochemical and histological analyses in these hearts show evidence of fibrosis and TGF-β pathway activation in concordance with our findings in hiPSC-CFs. Additionally, we identified BAG3-specific transcriptional changes in failing hearts, including increases in endoglin (*ENG*), a coreceptor for TGF-β. We suggest that these results are initial confirmation that BAG3 loss alters TGF-β signaling at a whole-organ level. However, our approach precluded study of TGFBR2 ubiquitination in these tissues. Whether TGFBR2 proteostasis is dysregulated within these patients requires additional study, though we note that the mRNA level of TGFBR2 did not significantly change compared with that in controls, suggesting post-transcriptional control. The dataset generated represents a valuable resource with the potential to study how BAG3 variants affect each cell type in the human heart.

Our study adds to the growing body of work using hiPSCs to study different cardiac cell types and their roles in maintaining cardiac homeostasis. These data underscore the ability of tissue engineering models to elucidate cell type–specific biology and the importance of using human explanted heart samples to define the cellular and histological phenotypic changes.

Apart from monogenic disease, BAG3 levels decrease in non-genetic heart failure, which affects a vastly larger number of patients. BAG3 is therefore a promising therapeutic target, as indicated by studies that have shown the preclinical efficacy of BAG3 overexpression in cardiac injury (83). The data presented here have implications for this approach, as we suggest that reconstitution of BAG3 in CMs alone may incompletely ameliorate the dysfunction caused by a global loss of BAG3. Conversely, because of the deleterious effect of BAG3 loss in both cell types, BAG3 may be a prime target to treat both CM pathology and fibroblast pathology, which rarely present without the other.

In conclusion, BAG3 emerges as a nexus of proteostasis crucial to not only cardiomyocytes, but also cardiac fibroblasts. These results broaden the knowledge of *BAG3* in the heart beyond a cardiomyocyte-centric view and highlight the ability of hiPSC-based cell type–specific modeling to help answer such questions.

### Study limitations.

Our study is not without limitations. We studied a homozygous knockout cell line, a severe and overrepresentative phenotype. Here, the majority of the *BAG3* gene, from exon 2, was deleted (26). This removes the functional domains of BAG3, including its IPV, PxxP, and BAG domains. We did not study which domain of BAG3 is responsible for the binding of TGFBR2. Additionally, as BAG3 does not have intrinsic E3 ubiquitin ligase activity, identifying the definitive E3 ligase that BAG3 facilitates to ubiquitinate TGFBR2 requires additional study. Furthermore, significant sex-specific differences in clinical survival were observed in patients with BAG3 DCM (3), and our data are limited to a single set of isogenic male hiPSCs.

## Methods

### Sex as a biological variable.

Our study examined 3 male and 1 female human patients with BAG3 pathogenic variants. Owing to the rarity of BAG3 variants, we did not obtain adequate sample sizes to conduct sex-specific analyses.

### hiPSC culture.

The male WT and *BAG3^–/–^* WTC11 hiPSC lines (26) were provided by Bruce Conklin, through a material transfer agreement between Gladstone Institutes and Columbia University. hiPSCs were cultured in mTeSR Plus medium (Stemcell Technologies) on plates coated with Matrigel (Corning 354230) until 70% confluence, then passaged using 0.5 mM EDTA every 4–6 days, with 5 μM ROCK inhibitor (RI; Tocris Y-27632) for the first day after passaging.

### CM differentiation.

Two days before differentiation (day –2), hiPSCs were replated in 6-well plates at a density of 2 million cells per well with 5 μM RI. The medium was refreshed with mTeSR Plus the following day (day –1). At the start of differentiation (day 0), the medium was switched to a cardiac differentiation medium (CDM) containing RPMI 1640, albumin, and ascorbic acid, supplemented with 6 μM CHIR 99021 (Tocris) (84). On day 2 after differentiation, medium was switched to CDM containing 2 μM Wnt-C59 (Tocris). Cells were refreshed in CDM on days 4, 6, and 8 after differentiation. At day 10 after differentiation, beating CMs were switched to RPMI 1640 plus B27 Supplement (Thermo Fisher Scientific), and were expanded one passage with the addition of 2 μM CHIR 99021 (Tocris), after dissociation with 10× TrypLE (Thermo Fisher Scientific) per a recently published method (85). Confluent CMs were maintained in RPMI 1640 plus B27 for experiments in 2D or until incorporation into tissues.

### Cardiac fibroblast differentiation and culture.

CFs were differentiated using an adapted version of an established protocol (16). Cardiac progenitor cells (CPCs) were generated using small-molecule modulation of Wnt signaling pathway in CDM medium, identical to CM differentiation, using Wnt-C59 rather than IWR2 as described in the original protocol. On day 5 after differentiation, CPCs were dissociated using Accutase (Stemcell Technologies) and split 1:12 in Matrigel-coated plates and Advanced DMEM (Thermo Fisher Scientific) supplemented with 2 μM retinoic acid, 5 μM CHIR, and 5 μM RI. On day 6, medium was refreshed to Advanced DMEM plus 5 μM CHIR and retinoic acid for 2 days. On day 8, cells were recovered in Advanced DMEM. On days 11–14, medium was switched to Advanced DMEM plus 2 μM SB431542 (Tocris). On day 14, cells were split at a density of 10,000/cm^2^ into Fibroblast Growth Medium 3 (FGM3; Promocell) supplemented with 20 ng/mL FGF-2 (PeproTech) and 10 μM SB431542 until day 20. Differentiated CFs were maintained in FGM3. For stiffness studies, cells were cultured atop 8 kPa Cytosoft plates (Advanced Biomatrix) that had been coated with Matrigel for 1 hour at room temperature.

Primary human ventricular cardiac fibroblasts (Lonza CC-2904) were cultured in FGM3 as well and were used at passage 3–5.

### Generation of cardiac tissues.

Tissues were generated and analyzed following our recently reported pipeline (19). Briefly, culture platforms were cast from polydimethylsiloxane in custom-milled molds containing electrodes for electrical stimulation. hiPSC-derived CMs and CFs were dissociated to single cells using 10× TrypLE (Thermo Fisher Scientific) and 1× TrypLE, respectively. Cells were resuspended to 500,000 cells per tissue in a ratio of 85% CMs and 15% CFs, in a solution of 5 mg/mL fibrinogen. 13.5 μL of cell suspension was mixed with 3 μL of thrombin (5 U/mL) in each well per tissue. After formation, tissues were maintained in fresh B27 medium. Seven days after tissue formation, medium was changed to a 50:50 mixture of B27 medium and an RPMI-based metabolic maturation medium. This culture medium contained AlbuMAX, a higher calcium content, and a lower glucose content designed to promote fatty acid oxidation, as detailed in a previous publication (86). Also at day 7, tissues began a 2-week ramped electrical stimulation regimen that started at 2 Hz and increased 0.33 Hz every 24 hours until 6 Hz. The medium was supplemented with 5 mg/mL 6-aminocaproic acid (Sigma-Aldrich A7824) throughout the tissue experiment.

### Measurement of cardiac tissue function.

Tissue contraction was captured by video-based microscopy while stimulated at 1 Hz, and force generation was analyzed by pillar deflection using a custom-written Python code, as recently reported (19). Briefly, a computer vision package containing an object-tracking algorithm was adapted to track pillar head movement and calculate displacement from videos of beating tissues. Cardiac tissue thickness was calculated from measurements in ImageJ (NIH) at the center of the tissue. Work was calculated by integration of the total force over the deflection distance of the pillar.

### Cardiac tissue staining.

Cardiac tissues were fixed using 4% paraformaldehyde (PFA), subsequently embedded in Histogel (Thermo Fisher Scientific), and fixed overnight in 4% PFA. The Histogel blocks were paraffin-embedded and sectioned along the longitudinal axis onto glass slides. Sections were rehydrated using washes in graded ethanol. Antigen retrieval was performed using 10 mM sodium citrate buffer, and the slides were microwaved until boiling. Sections were permeabilized with 0.25% Triton X-100 in PBS. Tissues were incubated with primary antibodies overnight, then secondary for 1 hour at room temperature in the dark before mounting on coverslips using ProLong Glass Antifade Mountant with NucBlue Stain (Invitrogen P36981), then imaged on a Nikon A1 confocal microscope. Mean gray value intensity of collagen deposition was quantified using ImageJ (87).

### Global proteomics through mass spectrometry.

For global quantitative proteomics of *BAG3^+/+^* and *BAG3^–/–^* CFs, proteomics based on parallel accumulation–serial fragmentation combined with data-independent acquisition (diaPASEF) was used (88). Briefly, cells were lysed in lysis buffer (1% Sodium deoxycholate (SDC), 100 mM Tris-HCl [pH 8.5], protease inhibitors) and boiled for 15 minutes at 95°C. Protein reduction and alkylation of cysteines was performed with 10 mM Tris(2-carboxyethyl)phosphine hydrochloride (TCEP) and 40 mM 2-Chloroacetamide (CAA) at 45°C for 10 minutes followed by sonication in a water bath cooled down to room temperature. Protein digestion was processed overnight by addition of LysC and trypsin at a 1:50 ratio (micrograms of enzyme to micrograms of protein) at 37°C and 1,400 rpm. Peptides were acidified by addition of 1% trifluoroacetic acid (TFA), vortexed, and subjected to StageTip (CDS Analytical) clean-up via styrenedivinylbenzene-reverse phase sulfonate (SDB-RPS) (89). Peptides were loaded on one 14-gauge StageTip plug. Peptides were washed twice with 200 μL 1% TFA/99% ethyl acetate followed by 200 μL 0.2% TFA/5% acetonitrile (ACN) in a centrifuge at 956*g*, followed by elution with 60 μL of 1% ammonia/50% ACN into Eppendorf tubes, and dried at 45°C in a SpeedVac centrifuge. Samples were resuspended in 10 μL of LC buffer (3% ACN/0.1% formic acid). Peptide concentrations were determined using a NanoDrop spectrophotometer (Thermo Fisher Scientific), and 200 ng of each sample was used for diaPASEF analysis on a timsTOF Pro (Bruker Daltonics). Peptides were separated within 120 minutes at a flow rate of 400 nL/min on a reversed-phase C18 column with an integrated CaptiveSpray Emitter (25 cm × 75 μm, 1.6 μm; IonOpticks). Mobile phases A and B were with 0.1% formic acid in water and 0.1% formic acid in ACN. The fraction of B was linearly increased from 2% to 23% within 90 minutes, followed by an increase to 35% within 10 minutes and a further increase to 80% before re-equilibration. The timsTOF Pro was operated in diaPASEF (88) mode, and data were acquired at defined 32 × 50 Th isolation windows from *m*/*z* 100 to 1,700. To adapt the MS1 cycle time in diaPASEF, the repetitions were set to 1.91 in the 32-scan diaPASEF scheme. The collision energy was ramped linearly as a function of the mobility from 59 eV at 1/K0 = 1.6 V·s/cm^2^ to 20 eV at 1/K0 = 0.6 V·s/cm^2^. The acquired diaPASEF raw files were searched with the default settings for targeted analysis of data-independent acquisition (DIA) data against the human UniProt database (UP000005640) in Spectronaut (90). The FDR was estimated with the mProphet approach (Biognosys AG) and set to 1% at peptide precursor level and at 1% at protein level. Results obtained from Spectronaut were further analyzed using the Spectronaut statistical package.

### Gene set enrichment analysis.

Gene set enrichment analysis (GSEA) was performed in the GSEA Java application (4.10.0). All protein abundances from the mass spectrometer were input. Proteins were filtered such that all 6 samples must have had detectable protein abundances. GSEA was run on the Hallmark gene set using gene set permutation with an FDR of 0.05.

### Analysis of global mass spectrometry data.

The FDR was estimated with the mProphet approach and set to 1% at peptide precursor level and at 1% at protein level. Results were further analyzed using the Spectronaut statistical package. Significantly changed protein abundance was determined by unpaired, 2-tailed *t* test with a threshold for significance of *P* less than 0.05 (permutation-based FDR correction) and 0.58 log_2_FC. Network analysis of differentially expressed proteins was performed in Cytoscape (v3.8.2) (https://cytoscape.org).

### Western blot.

Cells were washed with cold PBS and lysed using Pierce IP Lysis Buffer (Thermo Fisher Scientific 87787) containing protease and phosphatase inhibitors (Thermo Fisher Scientific 78442). Lysate was transferred to ice for 30 minutes with intermittent vortexing, homogenized by passing through a 26.5-gauge needle, then centrifuged at 16,000*g* for 10 minutes. Lysates were quantified with the Pierce BCA kit, and 20 μg total protein was mixed with 4× Laemmli buffer, boiled for 5 minutes, and loaded in 4%–20% Tris-glycine gels (Thermo Fisher Scientific XP04205BOX). Next, the gel was transferred onto 0.2 μm nitrocellulose membranes and blocked in 5% nonfat dry milk. Incubation with primary antibodies (see Supplemental Methods for catalog numbers) was done overnight at 4°C in 5% BSA or 5% milk in TBST, then washed and blotted with rabbit secondary antibody (1:5,000) in milk for 1 hour at room temperature. Membranes were imaged after incubation in enhanced chemiluminescence substrate (ECL).

For blots involving the detection of endogenous TGFBR2, whole-cell lysates were prepared by lysing of cells in 1% sodium dodecyl sulfate in 10 mM Tris-HCl buffer containing 1 mM sodium orthovanadate and boiling for 20 minutes. Samples were homogenized by passing through a 26.5-gauge needle 10 times. Samples were then spun at 16,000*g* for 10 minutes before transferring of cleared supernatant to a new tube. Sample protein concentration was determined via BCA assay, then mixed with 4× Laemmli buffer. At least 30 μg of sample was run under denaturing conditions in 4%–20% Tris-glycine gels (Thermo Fisher Scientific XP04205BOX), then transferred to 0.2 μm nitrocellulose membranes. Membranes were blocked in 5% nonfat dry milk. TGFBR2 antibody (1:250; Thermo Fisher Scientific 701683) was incubated overnight in TBS with 0.1% Tween-20, then washed and blotted with rabbit secondary (1:5,000) for 1 hour and imaged. Western blot membranes were imaged on a Licor Odyssey Fc system, and band intensity was quantified in ImageStudio Lite Software (v5.2).

### Transfection of plasmids and siRNA.

DNA constructs of FLAG-tagged BAG3 and V5-tagged TGFBR2 used in this study were purchased from and cloned by VectorBuilder (www.vectorbuilder.com). siRNA to TGFBR2 (Thermo Fisher Scientific s14077) and BAG3 (Thermo Fisher Scientific s18292) were transfected using Lipofectamine RNAiMAX following the manufacturer’s protocol. DNA plasmids were transfected using Viafect (Promega) following the manufacturer’s protocol with a ratio of 6:1 transfection reagent to plasmid (vol/wt). For ubiquitination assays involving V5-TGFBR2, pilot studies were performed to identify equivalent DNA concentrations for transfection, because of the increased stability of the protein in knockout cells. All plasmids used in this study can be found in the Supplemental Methods.

### Luciferase assay.

One day before the assay, cells were seeded in 24-well plates at a density of 50,000 cells per well. siRNAs were transfected as described. Twenty-four hours later, cells were transfected with SBE4-Luc (Addgene 16495) and pRL (Promega E2261) at a 1:1 ratio using Viafect as described. Twenty-four hours after transfection, medium was refreshed with or without 10 ng/mL TGF-β1 (PeproTech 100-21C). Twenty-four hours later, luciferase activity was analyzed using the Dual-Glo Luciferase Assay Kit (Promega E2920) following the manufacturer’s instructions.

### Quantitative reverse transcriptase PCR.

mRNA was isolated from cells in a 12-well plate using the RNeasy Mini Kit (QIAGEN 74106) according to the manufacturer’s instructions. RNA quality was ensured with 260:230 ratio between 1.8 and 2.2 using the NanoDrop One (Thermo Fisher Scientific). cDNA samples were obtained from 1 μg RNA using a High Capacity cDNA Reverse Transcription Kit (Applied Biosystems, catalog 4368813). Reverse transcriptase quantitative PCR (RT-qPCR) was performed with 5 ng cDNA per sample, 10 μM of forward and reverse primer (Supplemental Methods), and Fast SYBR Green Master Mix (Applied Biosystems, catalog 4385612) in a 96-well plate (Applied Biosystems, catalog 4346906). Thermal cycling was performed in a StepOne Plus Real Time PCR System (Applied Biosystems) using 40 cycles of 2-step PCR protocol of 95°C melting for 3 seconds and 60°C annealing and extension for 30 seconds, before an initial hot start at 95°C for 20 seconds.

### Affinity purification mass spectrometry.

Eluted proteins from FLAG immunoprecipitation reactions were denatured in SDC buffer (0.5% SDC/100 mM Tris-HCl [pH 8.5]) and boiled for 20 minutes at 60°C, 1,500 rpm (89). Protein reduction and alkylation of cysteines were performed with 10 mM TCEP and 40 mM CAA at 45°C for 10 minutes followed by sonication in a water bath cooled down to room temperature. Proteins were further precipitated with salt method as previously described, and pellets were resuspended in SDC buffer (1% SDC/100 mM Tris-HCl [pH 8]) (89). Protein digestion was processed overnight by addition of LysC and trypsin at a 1:50 ratio (micrograms of enzyme to micrograms of protein) at 37°C and 1,400 rpm. Peptides were acidified by addition of 1% TFA, vortexed, and subjected to StageTip clean-up via SDB-RPS and dried in a SpeedVac. Peptides were dissolved in 3% ACN/0.1% formic acid. Peptides were separated within 80 minutes at a flow rate of 400 nL/min on a reversed-phase C18 column with an integrated CaptiveSpray Emitter (25 cm × 75 μm, 1.6 μm; IonOpticks). Mobile phases A and B were with 0.1% formic acid in water and 0.1% formic acid in ACN. The fraction of B was linearly increased from 2% to 23% within 70 minutes, followed by an increase to 35% within 10 minutes and a further increase to 80% before re-equilibration. The timsTOF Pro was operated in PASEF (91) mode with the following settings: mass range 100 to 1,700 *m*/*z*, 1/K0 start 0.6 V·s/cm^2^, end 1.6 V·s/cm^2^, ramp time 100 ms, lock duty cycle to 100%, capillary voltage 1,600 V, dry gas 3 L/min, dry temperature 200°C; PASEF settings: 10 tandem mass spectrometry (MS/MS) frames (1.16 seconds duty cycle), charge range 0–5, active exclusion for 0.4 minutes, target intensity 20,000, intensity threshold 2,500, collision-induced dissociation (CID) collision energy 59 eV. A polygon filter was applied to the *m*/*z* and ion mobility plane to select features most likely representing peptide precursors rather than singly charged background ions.

### Analysis of liquid chromatography–tandem mass spectrometry data (AP-MS).

Acquired PASEF raw files were analyzed using the MaxQuant environment v2.0.1.0 and Andromeda for database searches at default settings (92). The default is used for first search tolerance and main search tolerance (20 ppm and 4.5 ppm, respectively). MaxQuant was set up to search with the reference human proteome database (UP000005640) downloaded from UniProt. MaxQuant performed the search trypsin digestion with up to 2 missed cleavages. Peptide, site, and protein FDRs were all set to 1% with a minimum of 1 peptide needed for identification; label-free quantitation was performed with a minimum ratio count of 1. The following modifications were used for protein identification and quantification: Carbamidomethylation of cysteine residues (+57.021 Da) was set as a static modification, while the oxidation of methionine residues (+15.995 Da) and deamidation (+0.984) on asparagine and glutamine were set as a variable modifications. Results obtained from MaxQuant were imported into Perseus v1.6.10.0 (93) for 2-tailed *t* test statistical analysis (FDR < 0.01) to identify proteins demonstrating statistically significant changes in abundance.

### Immunoprecipitation.

Cells were lysed in a custom buffer (MS lysis buffer) containing 0.5% (vol/vol) NP-40 detergent, 150 mM sodium chloride, 10 mM potassium chloride, 1.5 mM magnesium chloride, and 10 mM Tris-HCl buffer supplemented with protease and phosphatase inhibitors. Lysate was transferred to ice for 30 minutes with intermittent vortexing, then centrifuged at 16,000*g* for 10 minutes at 4°C. The supernatant was removed to a clean tube and protein concentration quantified. Each immunoprecipitation reaction contained 300–500 μg of equivalent protein. For endogenous BAG3 immunoprecipitation, 5 μg of BAG3 antibody (Proteintech 10599-1AP) was incubated with Dynabeads Protein G (25 μL) (Thermo Fisher Scientific) in TBS with 0.1% Tween-20 (TBST) for 30 minutes at room temperature. Antibodies were cross-linked to beads per the manufacturer’s instructions using bis(sulfosuccinimidyl)suberate (BS_3_; Thermo Fisher Scientific 21580). Cross-linked beads were washed 3 times with TBST, then incubated in lysate overnight at 4°C. For other immunoprecipitation reactions, FLAG-conjugated (Sigma-Aldrich M8823) and V5-conjugated (Chromotek v5tma) magnetic beads were used, according to the manufacturers’ protocols. After sample incubation, beads were washed with MS lysis buffer 10 times; then sample was eluted depending on downstream application. For mass spectrometry, beads were incubated in 0.1 M glycine-HCl (pH 2.8) for 30 minutes at room temperature, then quenched in 1 M Tris-HCl. For Western blotting, samples were eluted from the beads using 2× Laemmli buffer for 5 minutes at 95°C.

### Chase studies.

Cells were replated at a density of 50,000 cells/cm^2^ 2 days prior to the start of treatment. Cells were treated with 50 μg/mL cycloheximide (Sigma-Aldrich C4859), 50 μM bafilomycin A1 (Tocris 1334), or 10 μM MG-132 (Selleck S2619) for the specified times and subsequently collected and lysed for protein analysis by Western blot.

### Flow cytometry.

Dissociated and pelleted single cells were fixed in 4% PFA. Cells were washed twice with PBS and permeabilized using 0.1% Triton X in PBS before incubation with primary antibody solution for troponin (BD Biosciences 565744) per the manufacturer’s protocol. After staining for 30 minutes, cells were washed 3 times with PBS and analyzed on a Novocyte Quanteon cell analyzer. EdU uptake was measured using Click-iT EdU Cell proliferation assays (Thermo Fisher Scientific) according to the manufacturer’s protocol and measured using flow cytometry in the Novocyte Quanteon cell analyzer.

### Immunofluorescence quantification of proliferation.

CFs were thawed on 96-well imaging plates (Corning CLS4580) at a density of 50,000 cells per well. Forty-eight hours later, cells were fixed using 4% PFA and permeabilized with 0.1% Triton-X in PBS at room temperature. Cells were blocked in 5% BSA for 1 hour, then incubated in primary antibody against Ki-67 overnight (Abcam). The following day, cells were incubated with secondary antibody for 1 hour followed by 3 PBS washes. Then, cells were stained with phalloidin and DAPI for 15 minutes, followed by 3 PBS washes. Cells were then imaged on a Molecular Devices ImageXpress Pico. Image analysis was conducted in an automated manner using CellProfiler (https://cellprofiler.org). Briefly, nuclei were identified in the DAPI channel. From the identified nuclei, the Ki-67 channel was masked and the percentage of nuclei containing Ki-67 calculated.

### Streptavidin pull-down.

CFs were seeded at a density of 3 million per 10 cm dish. Cells were grown for 4 days until confluence. The Pierce Cell Surface Pulldown Kit was used per manufacturer’s protocol to biotinylate and pull down cell surface proteins. An equivalent amount of 800 μg protein was used per pull-down reaction, and cell lysate was incubated with streptavidin beads overnight at 4°C. The eluted lysate was mixed with 4× Laemmli buffer and subsequently used for Western blot.

### Measurement of proteasomal activity.

CFs were lysed in Pierce IP Lysis Buffer (Thermo Fisher Scientific 87787) with protease and phosphatase inhibitors (as above) and quantified using BCA assay. Subsequently, 40 μg of lysate was loaded onto an opaque white 96-well plate, and proteasome activity was measured according to the manufacturer’s instructions with a commercially available proteasome activity assay (Abcam ab107921).

### Collagen secretion assay.

Supernatant of hiPSC-CFs cultured on soft 8 kPa plates with and without 10 ng/mL TGF-β1 for 48 hours of treatment was collected, and collagen was concentrated using a concentrating solution (Chondrex 90626) according to the manufacturer’s instructions. Secreted collagen was determined using Sirius Red Total Collagen Detection Kit (Chondrex 9062) according to the manufacturer’s instructions, and absorbance was measured at 530 nm in a spectrophotometer.

### Multiplexed single-nucleus RNA sequencing.

Cells were cultured as described earlier and then frozen. Multiplexing, single-nucleus RNA sequencing (snRNA-Seq), and deconvolution were conducted as previously described (53). CellBender (94) was used to remove unwanted background RNA reads. For doublet detection, Scrublet (95) and Solo (96) were used. Nuclei with Scrublet scores less than 0.3 and Solo scores less than 0.5 were included in subsequent analyses. Nuclei meeting the following quality control metrics were retained for further analyses: RNA count between 15,000 and 5,000; number of detected genes between 6,000 and 300; mitochondrial gene reads less than 5%; ribosomal gene reads less than 5%. Counts were normalized and scaled using the standard Seurat commands (97). Mitochondrial percentages and RNA counts were regressed out. Nuclei were clustered at a resolution of 0.1. For differential gene expression testing, genes with a normalized averaged expression greater than 0.2 in at least one genotype were used (*n* = 9,236). Differentially expressed genes (DEGs) were tested using edgeR (98). The results were filtered based on an adjusted *P* value (<0.05) and an absolute log_2_ fold change (1). Significant DEGs are listed in Supplemental Table 4. Gene Ontology analysis of the DEGs was performed using the ClusterProfiler package (99), with the 9,236 genes as the background. *P* values were adjusted using the Bonferroni method, and all ontology databases were tested. The hypergeometric test (phyper) was used to calculate significant overlaps between the DEGs. For the hypergeometric test of proteomics and the snRNA-Seq data, genes detected and tested in both platforms (*n* = 4,739) were used.

### Human tissue snRNA-Seq analysis.

Tissues were sequenced and processed as previously described (28, 100). All sequencing data were processed using CellBender (94) to eliminate nonspecific RNA expression. Samples were then merged into a single Seurat object. Solo (96) was used for doublet detection. Nuclei meeting the following quality control metrics were retained for subsequent analyses: RNA count between 15,000 and 5,000; detected genes between 6,000 and 300; mitochondrial gene reads below 1%; ribosomal gene reads below 1%. To remove batch effects and unwanted variations, samples were harmonized based on individuals (101). Gene expression was normalized using standard Seurat commands, and nuclei were clustered at a resolution of 0.1. Fibroblasts were then isolated, renormalized, and clustered using Leiden clustering in the Scanpy package (102). Fibroblast states were annotated based on the previous study (28). The abundance of each cell type and fibroblast states are listed in Supplemental Table 5. Then tissues were grouped into control (*n* = 14), BAG3 (*n* = 4), and DCM (DCM caused by mutations in *TTN* or *LMNA*, *n* = 9 for each) for statistical testing. Distribution of nuclei among different states was tested using 2-tailed Student’s *t* test and adjusted for multiple testing using the Bonferroni method. For differential gene expression analysis, genes with a normalized average expression greater than 0.0125 in at least one genotype group were selected (*n* = 18,798) and analyzed using edgeR (98). For differential gene expression analysis within each state, samples with less than 10 nuclei in a given state were excluded from the analysis. DEGs with an adjusted *P* value less than 0.05 and an absolute fold change greater than 1.5 were reported. Significant DEGs are listed in Supplemental Table 4.

### Histological analysis.

Cross sections of hearts were fixed in 10% formalin and embedded in paraffin. Sections were sliced to a thickness of 5 μm from the block and stored at 4°C. Before staining, the tissue sections were deparaffinized with ethanol and xylene, then rehydrated according to the protocol. Slices were stained with Picrosirius red and Masson’s trichrome to assess fibrosis, which was visualized using a light microscope (Leica DM4000 B), as described in our previous research (28, 103). Five sections per sample were imaged, with 7 pictures taken per section. The area of collagen deposition was quantified using ImageJ (Fiji) by selection of the area and adjustment of the color threshold. Raw data are provided in Supplemental Table 6.

### Statistics.

GraphPad Prism version 9.3.1 was used for all statistical analyses. The details of statistical analysis, including *n*, test used, and significance, are shown in figures and figure legends. All experiments reported are representative of at least 3 independent experiments, each performed with at least 3 independent cardiac fibroblast differentiations.

### Study approval.

Patients with advanced HF were recruited at Columbia University Medical Center, and heart tissue at the time of heart transplant was collected from the left ventricles of explanted hearts. Explanted heart samples from the Mazankowski Alberta Heart Institute (Edmonton, Alberta, Canada) were collected via the Human Explanted Heart Program (HELP; Pro00011739).

Control myocardial samples were obtained from the National Disease Research Interchange (https://ndriresource.org/ats) as deidentified specimens collected from non-failing hearts determined to be unusable for cardiac transplantation due to non-cardiac donor issues but without evidence or knowledge of underlying cardiac disease. The study was approved by the Institutional Review Board of Columbia University (IRB no. AAAR0055). All patients provided written informed consent before inclusion in the study.

### Data availability.

The mass spectrometry proteomics data were deposited to ProteomeXchange via the PRIDE (104) partner repository with the dataset identifier PXD050740. snRNA-Seq data of hiPSC-CFs were deposited in the Gene Expression Omnibus database (GEO GSE261750). Original Western blots and raw data are available in the Supporting Data Values file. Any additional information required to reanalyze the data reported in this paper is available upon request. Python scripts used to analyze engineered tissues are available via the Vunjak-Novakovic laboratory’s GitHub page (https://github.com/GVNLab).

## Author contributions

BZW conceptualized the study. BZW, MAJM, and SWK designed experiments. BZW, MAJM, SWK, LJL, XZ, RKS, RIL, JR, AZ, Youngbin Kim, MN, JMG, Yuri Kim, KB, DMD, QZ, BM, and JMW conducted experiments and analyzed data. GYO and BMF provided patient samples. BZW and SWK wrote the initial draft. BZW, MAJM, LJL, RIL, XZ, KMC, BMF, JGS, CES, GYO, and GVN edited the manuscript. JGS, CES, GYO, BMF, and GVN supervised the study and approved the final manuscript. GVN, JGS, CES, and GYO acquired funding.

## Supplementary Material

Supplemental data

Unedited blot and gel images

Supplemental table 1

Supplemental table 2

Supplemental table 3

Supplemental table 4

Supplemental table 5

Supplemental table 6

Supporting data values

## Version 1 01/02/2025

Electronic publication
