# Supplementary material for: Cardiac fibroblast BAG3 regulates TGFBR2 signaling and fibrosis in dilated cardiomyopathy
Source: J Clin Invest. 2025 Jan 2;135(1):e181630. doi: 10.1172/JCI181630 (PMC11684812; doi:10.1172/JCI181630)
Supplement: Supplemental data [file jci-135-181630-s032.pdf]

Supplemental Materials

Cardiac fibroblast BAG3 regulates TGFBR2 signaling and fibrosis in dilated cardiomyopathy

Bryan Z. Wang[1†], Margaretha A.J. Morsink[1†], Seong Won Kim[2], Lori J. Luo[1], Xiaokan Zhang[3], Rajesh Kumar Soni[4,5], Roberta I. Lock[1], Jenny Rao[3], Youngbin Kim[1], Anran Zhang[6,7], Meraj Neyazi[2,8,9], Joshua M. Gorham[2], Yuri Kim[2,10], Kemar Brown[2,11], Daniel M DeLaughter[2], Qi Zhang[2], Barbara McDonough[10,12], Josephine M. Watkins[1], Katherine M. Cunningham[1], Gavin Y Oudit[6,7], Barry M. Fine[3], Christine E. Seidman[2,10,12], Jonathan G. Seidman[2], Gordana Vunjak-Novakovic[1,3]

Affiliations

1. Department of Biomedical Engineering, Columbia University, New York, NY, USA.
2. Department of Genetics, Harvard Medical School, Boston, MA, USA.
3. Department of Medicine, Columbia University Medical Center, New York, NY, USA.
4. Proteomics and Macromolecular Crystallography Shared Resource, Herbert Irving Comprehensive Cancer Center, Columbia University Irving Medical Center, New York, NY, USA.
5. Department of Pathology and Cell Biology, Columbia University Irving Medical Center, New York, NY, USA.
6. Division of Cardiology, Department of Medicine, Faculty of Medicine and Dentistry, University of Alberta, Edmonton, Alberta T6G 2R3, Canada.
7. Mazankowski Alberta Heart Institute, Faculty of Medicine and Dentistry, University of Alberta, Edmonton, Alberta T6G 2R3, Canada.
8. Department of Cardiology, University Heart & Vascular Center Hamburg, University Medical Center Hamburg-Eppendorf, Hamburg, Germany
9. German Centre for Cardiovascular Research (DZHK), Partner Site Hamburg/Kiel/Luebeck, Hamburg, Germany
10. Cardiovascular Division, Brigham and Women's Hospital, Boston, MA 02115, USA.
11. Cardiac Unit, Massachusetts General Hospital, Boston, MA 02114, USA.
12. Howard Hughes Medical Institute, Bethesda, MD 20815, USA.

[†] These authors contributed equally to this work.

Key words: Cardiology, Cardiovascular disease, Fibrosis, Human stem cells

This supplement contains:

Figures S1-S6

Supplemental Table 1: Global Proteomic analysis of BAG3KO and WT hiPSC-derived CFs

Supplemental Table 2: High confidence interactors of BAG3 in CFs

Supplemental Table 3: Patient characteristics of BAG3 pathogenic variant heart donors

Supplemental Table 4: Differential gene expression of sn-RNAseq between hiPSC-CFs as well as DCM versus healthy control.

Supplemental Table 5: Cell type and cell state abundance in sequenced hearts

Supplemental Table 6: Raw value table for histological quantification of fibrosis in human hearts

Supplemental Methods: list of key commercial products

Additional files:

Unedited blot and gel images: Raw uncropped westerns

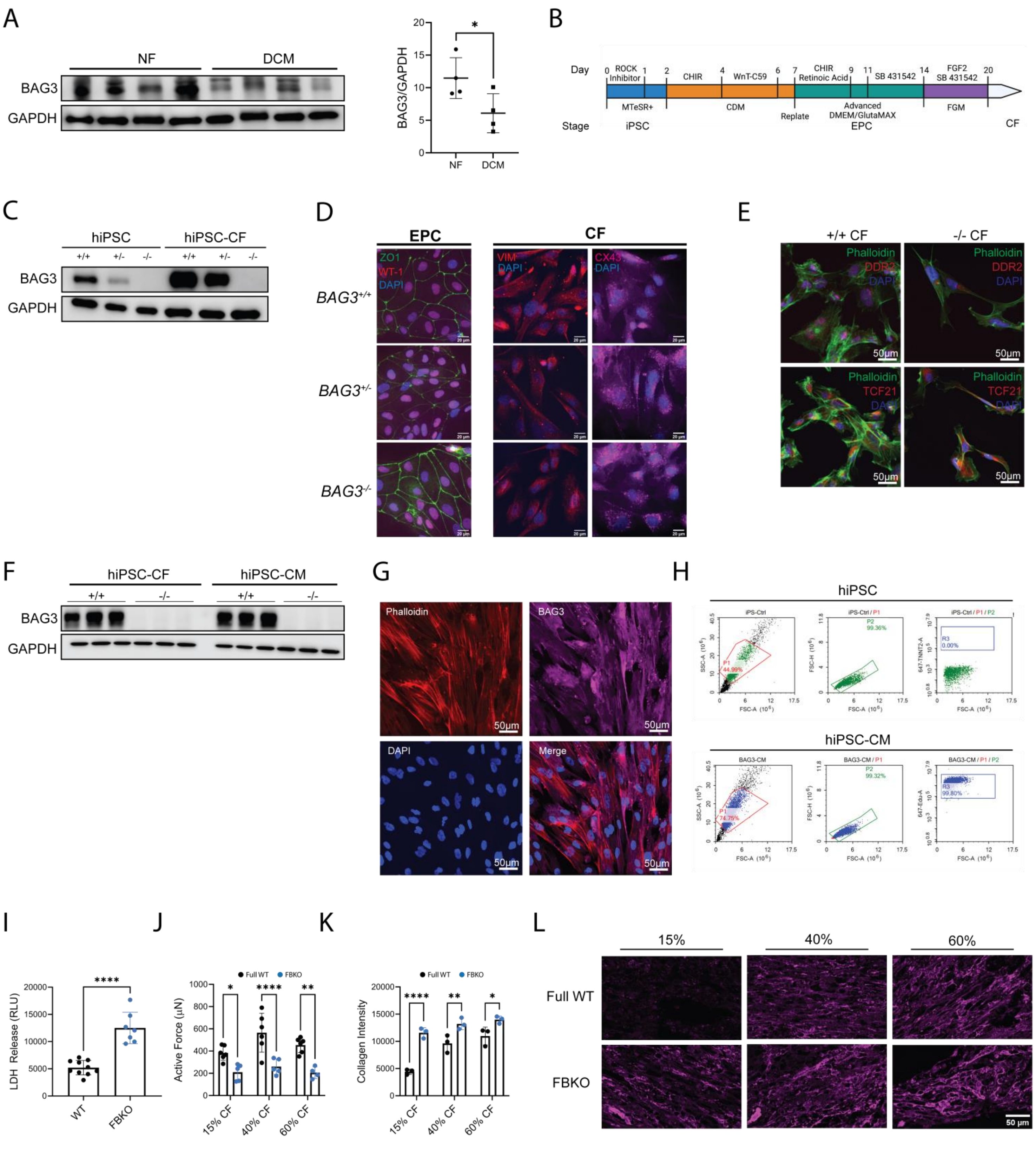
A
NF
DCM
BAG3
GAPDH
BAG3/GAPDH
*
NF
DCM
B
Day
0 ROCK Inhibitor 1
2 CHIR 4 WnT-C59 6
7 CHIR Retinoic Acid 9
11 SB 431542 14
FGF2 SB 431542 20
MTeSR+
CDM
Replate
Advanced DMEM/GlutaMAX
FGM
Stage
iPSC
EPC
CF
C
hiPSC
hiPSC-CF
+/+ +/- -/- +/+ +/- -/-
BAG3
GAPDH
D
EPC
CF
ZO1
WT-1
DAPI
VIM
DAPI
CX43
DAPI
BAG3+/+
BAG3+/-
BAG3-/-
E
+/+ CF
-/- CF
Phalloidin
DDR2
DAPI
TCF21
50μm
F
hiPSC-CF
hiPSC-CM
+/+ -/- +/+ -/-
BAG3
GAPDH
G
Phalloidin
BAG3
DAPI
Merge
50μm
H
hiPSC
hiPSC-CM
I
LDH Release (RLU)
****
WT
FBKO
J
Full WT
FBKO
Active Force (μN)
*
****
**
15% CF
40% CF
60% CF
K
Full WT
FBKO
Collagen Intensity
****
**
*
15% CF
40% CF
60% CF
L
15%
40%
60%
Full WT
FBKO
50 μm

Figure S1: **(A)** Loss of BAG3 in human dilated cardiomyopathy (DCM) heart lysates versus non-failing (NF) controls. **(B)** Schematic of hiPSC-CF differentiation protocol. **(C)** Validation of BAG3 heterozygous and homozygous knockout by Western Blot. **(D)** Differentiated hiPSC-CFs undergo an epicardial progenitor state before CF specification. **(E)** Immunofluorescence staining of cardiac fibroblast markers DDR2 and TCF21 in $BAG3^{+/+}$ and $BAG3^{-/-}$ hiPSC-CF. **(F)** BAG3 levels in hiPSC-CF versus hiPSC-CM by Western Blot. **(G)** Immunofluorescent localization of BAG3 in wild-type hiPSC-CF. **(H)** Representative measurement of TNNT2+ hiPSC-CMs by flow cytometry. **(I)** LDH release by Full WT and FBKO engineered cardiac tissues. **(J)** Active force generated by Full WT and FBKO engineered cardiac tissues of varying fibroblast composition. **(K)** Quantification and representative images (**L)** of collagen deposition in Full WT and FBKO engineered heart tissues of varying fibroblast composition. * = p<0.05, ** = p<0.01, **** = p < 0.001 by unpaired two-tailed student's t-test (A, I) or two-way ANOVA with Sidak's (J,K).

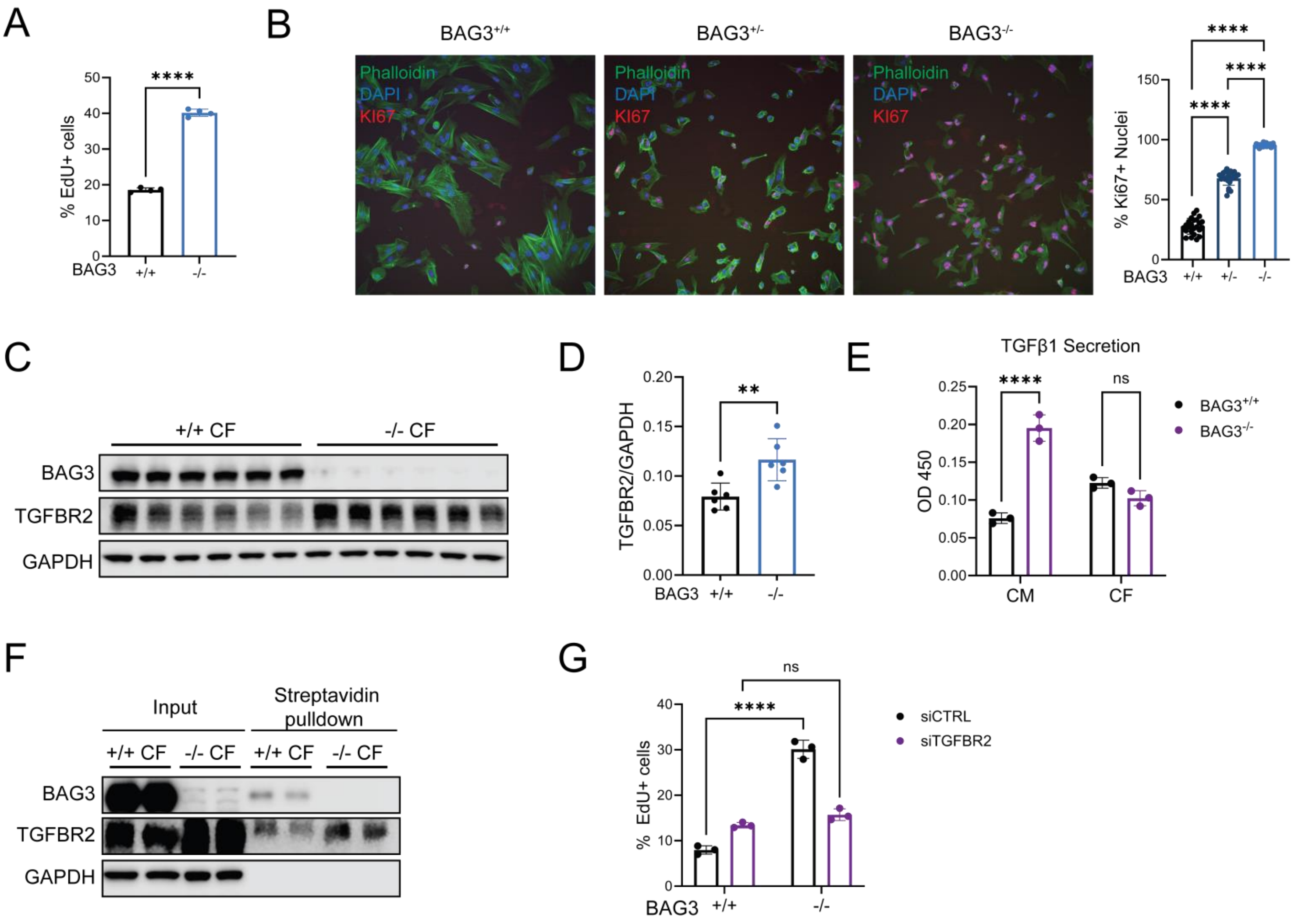

Figure S2: (A) Proliferation difference in hiPSC-CF quantified via EdU staining and flow cytometry. **(B)** Dose dependent increase in Ki67+ nuclei quantified across BAG3 knockout CFs. **(C and D)** Quantification of TGFBR2 levels shown by western blot. N = 6 from 3 independent differentiations. **(E)** TGFβ1 ligand secretion measured in the cell culture supernatant of hiPSC-CFs and hiPSC-CMs by ELISA. **(F)** Cell membrane biotinylation and streptavidin pulldown shows an increase in TGFBR2 at the membrane and colocalization with BAG3. **(G)** Silencing of TGFBR2 reduces the proliferative phenotype in *BAG3*$^{-/-}$ CFs measured by EdU uptake and flow cytometry. ** $p < 0.01$, **** $p < 0.0001$ by unpaired two-tailed student's t-test (A,D) or ANOVA with post-hoc Tukey's (one-way) (B) or Sidak's (two-way) (E,G).

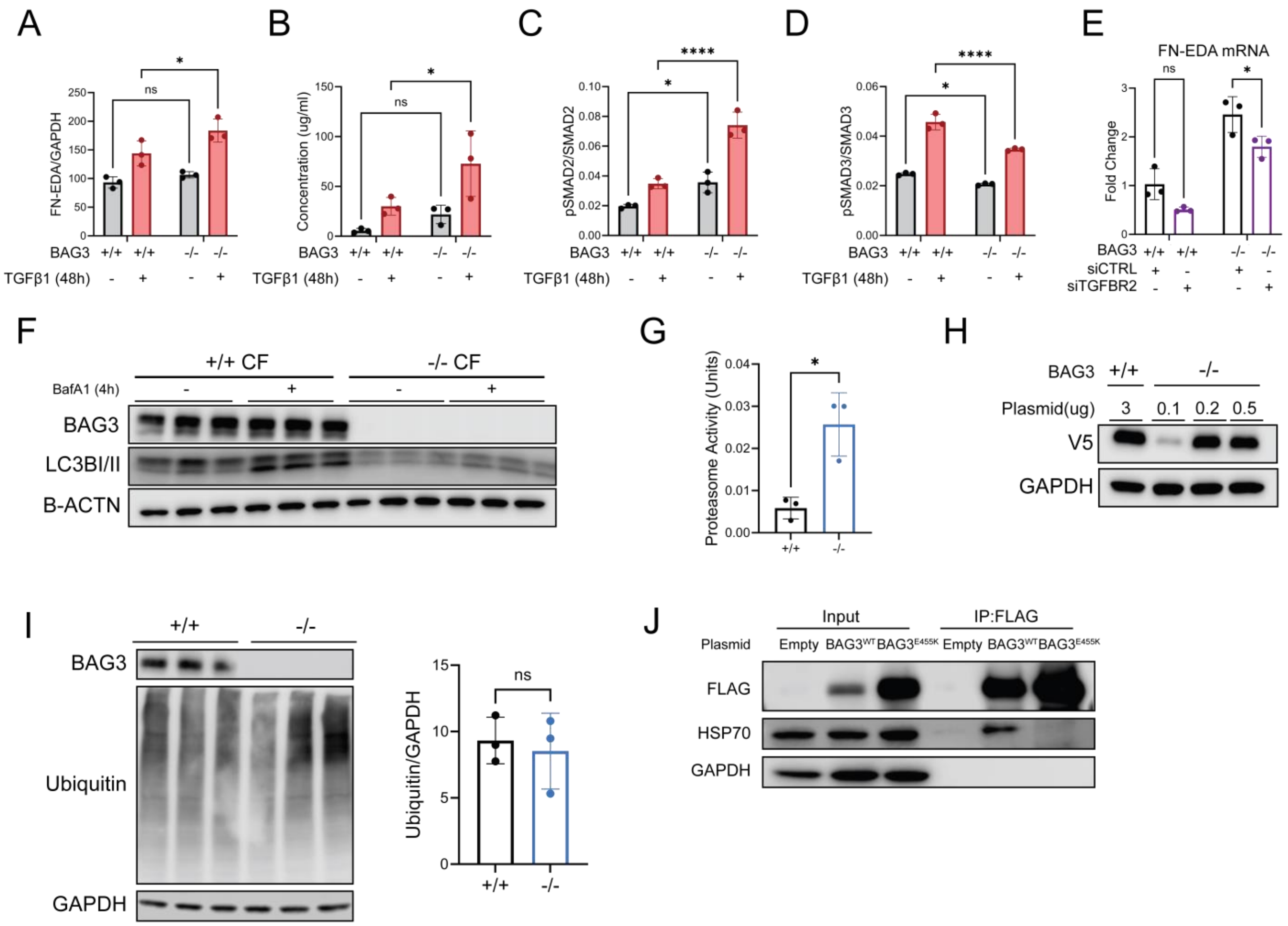

Figure S3. (A) Densitometry analysis of FN-EDA from Figure 3J. **(B)** Collagen secreted into the supernatant of hiPSC-CFs grown on 8kPa substrates with and without TGFβ ligand stimulation **(C)** Densitometry analysis of SMAD2 phosphorylation and **(D)** SMAD3 phosphorylation from Figure 3J. **(E)** Silencing of TGFBR2 reduces FN-EDA mRNA levels. **(F)** Loss of autophagic flux measured by LCBI/II levels. **(G)** Proteasome proteolytic activity measured using by cleavage of a fluorometric substrate. **(H)** Titration of V5-TGFBR2 transfection to obtain equivalent pulldown. **(I)** Comparison of global ubiquitination in BAG3KO and WT hiPSC-CFs. **(J)** FLAG-IP demonstrating the loss of HSP70-BAG3 interaction in BAG3^{E455K}. * $p < 0.05$, **** $p<0.0001$ by unpaired two-tailed student's t-test (G,I) or two-way ANOVA with post-hoc Sidak's (A-E).

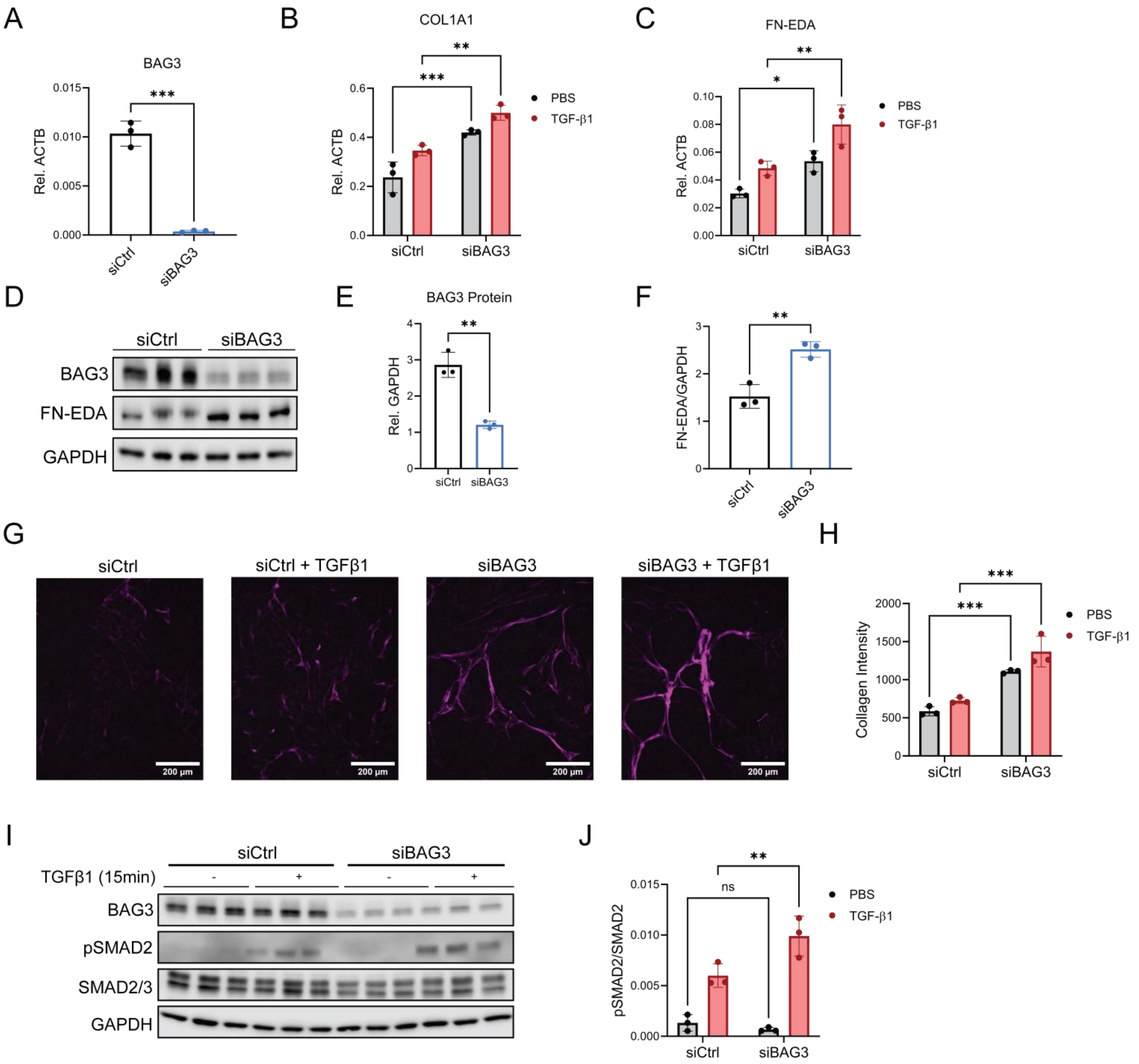

Figure S4. (A) RT-qPCR of BAG3 in human primary cardiac fibroblasts upon silencing of BAG3. **(B)** RT-qPCR analysis of COL1A1 gene expression in human primary CFs upon silencing of BAG3 on 8kPa and exposure to TGF-β1 for 48h. **(C)** RT-qPCR analysis of FN-EDA gene expression in human primary CFs upon silencing of BAG3 on 8kPa and exposure to TGF-β1 for 48h. **(D)** Western blot of FN-EDA upon silencing of BAG3 in primary human cardiac fibroblasts. **(E)** Densitometry quantification of BAG3 and **(F)** FN-EDA levels. **(G)** Immunofluorescence of collagen 1 in primary cardiac fibroblasts in response to silencing of BAG3 at 8kPa and quantification in **(H)**. Each dot represents the average of the mean fluorescent intensity of 3

separate images per well. **(I)** Western blot of SMAD2 phosphorylation after 15 minutes of TGF-β1 ligand exposure upon BAG3 knockdown in primary human CFs cultured on TCP. **(J)** Densitometry analysis of SMAD2 phosphorylation. ** $p<0.01$, ***$p<0.005$, **** $p<0.0001$ by unpaired student's t-test (A,E,F) or two-way ANOVA with post-hoc Sidak's (B,C,H,J).

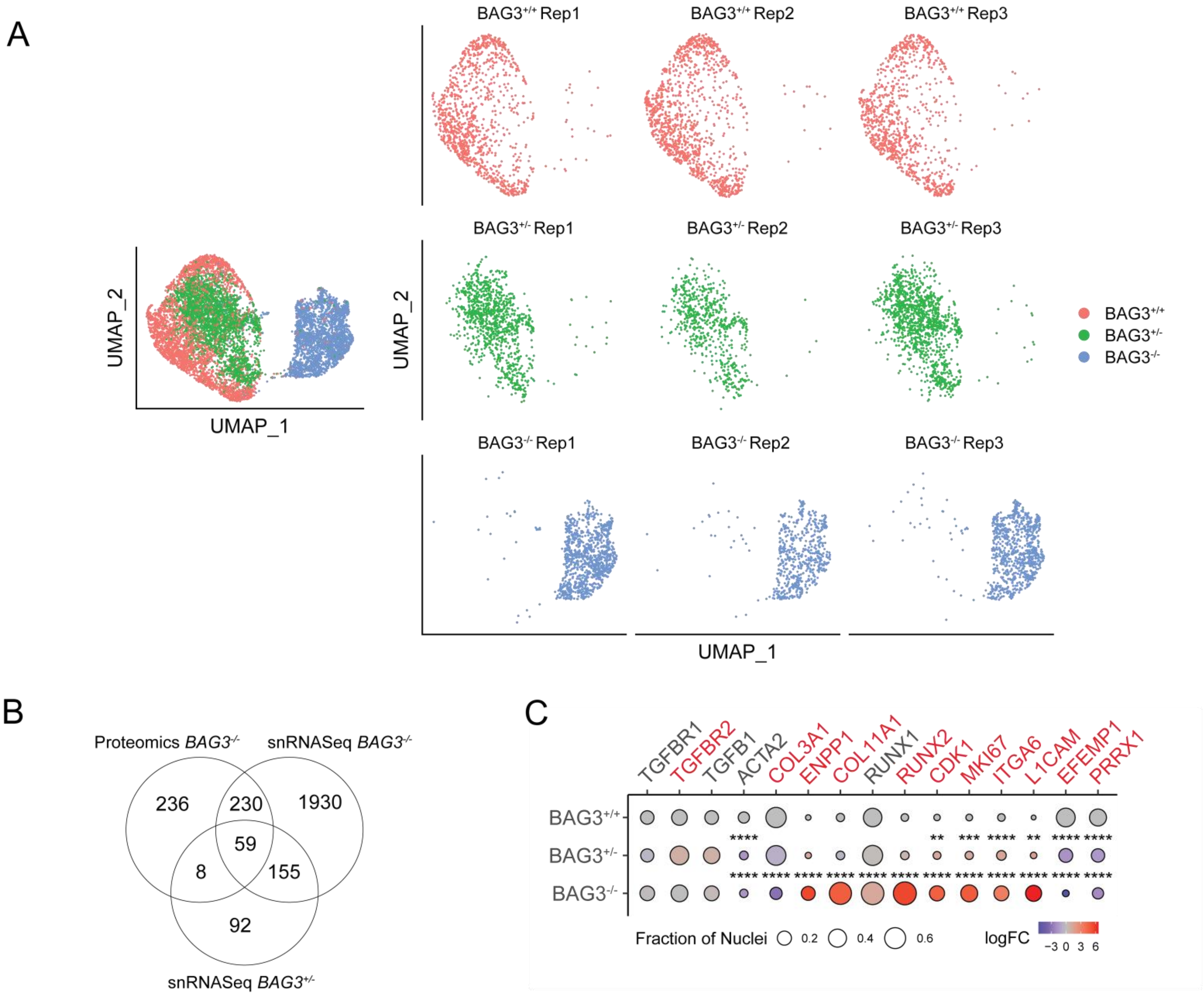

Figure S5. (A) UMAP highlighting each biological replicate of hiPSC-CFs. **(B)** Venn diagram illustrating the overlaps between DEGs and differentially expressed proteins. Genes tested in both platforms (N=4739) are displayed. **(C)** Dot plot depicting gene expression levels. Nuclei were grouped by their genotypes, and the color denotes the log2 fold change from wildtypes. The size of each dot indicates the percentage of nuclei expressing the respective gene. Genes differentially expressed in proteomics as well are highlighted in red. Adjusted P values from *EdgeR* analysis are provided at the top of each dot. **** P<0.0001; *** P<0.001; ** P<0.01; * P<0.05.

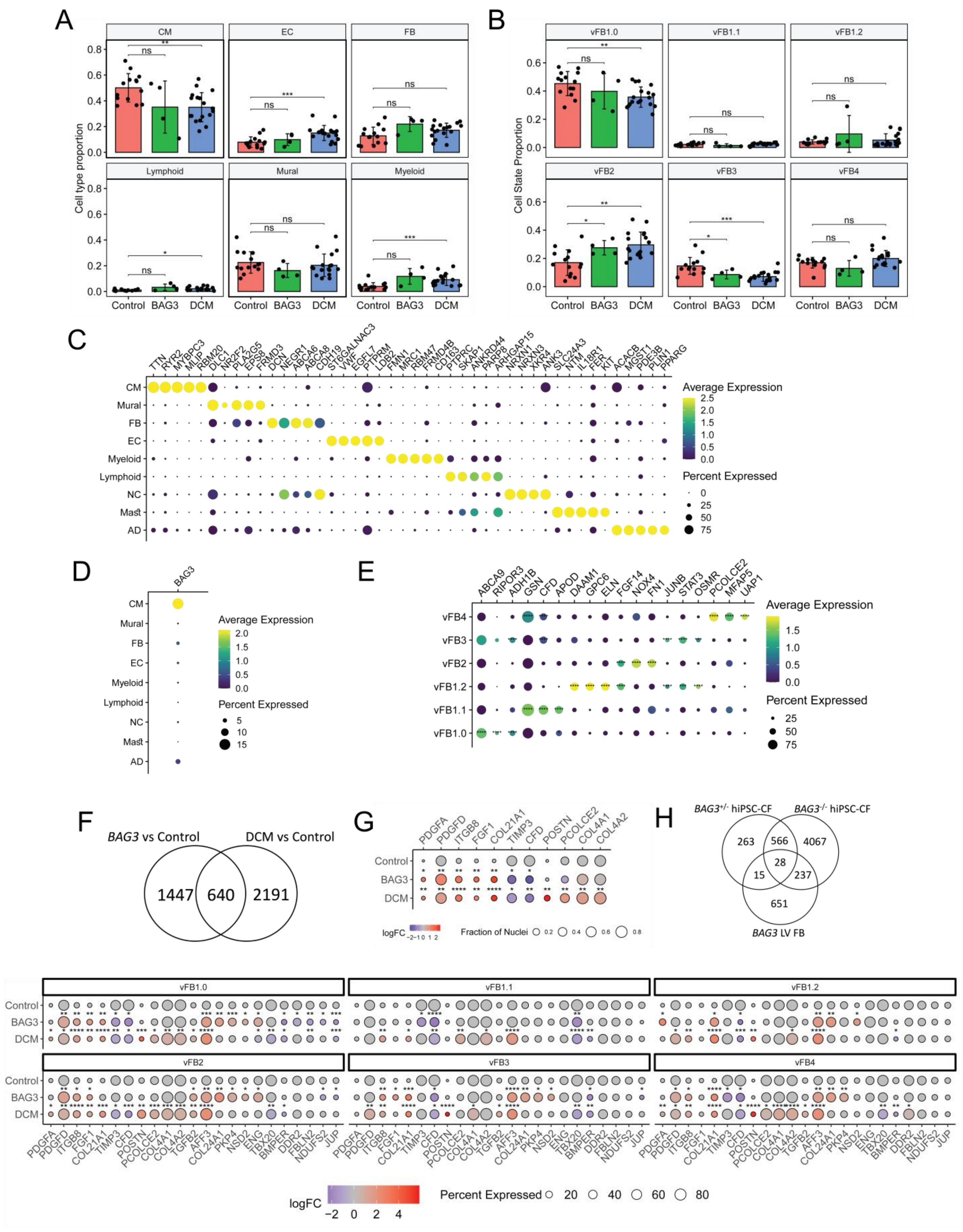

A
B
C
D
E
F
G
H
I
Cell type proportion
Cell State Proportion
CM
EC
FB
Lymphoid
Mural
Myeloid
vFB1.0
vFB1.1
vFB1.2
vFB2
vFB3
vFB4
Control
BAG3
DCM
ns
Average Expression
Percent Expressed
NC
Mast
AD
BAG3 vs Control
DCM vs Control
1447
640
2191
BAG3+/- hiPSC-CF
BAG3-/- hiPSC-CF
BAG3 LV FB
263
566
4067
28
15
237
651
logFC
Fraction of Nuclei

Figure S6. **(A)** Distribution of nuclei across the major cell types in LVs. **(B)** Distribution of fibroblast nuclei across different fibroblast states. For **(A)** and **(B),** P-values were calculated using a T-test and adjusted for multiple testing using Bonferroni correction. **(C)** Expression levels of known marker genes for each cell type. **(D)** Expression levels of *BAG3* across all cell types in LV. **(E)** Expression levels of marker genes for each fibroblast state. Stars indicate P-values calculated by Wilcoxon rank sum test and adjusted for multiple testing using Benjamini-Hochberg method. For C, D, and E, the size of each dot represents the percent of nuclei expressing the corresponding gene. Colors reflect normalized and scaled gene expression. **(F)** Venn diagram illustrating the overlap between the significant DEGs in *BAG3* and DCM fibroblasts. **(G)** Dot plot of pro-fibrotic genes. Nuclei were grouped by their genotypes, and the color denotes the log2 fold change compared to nonfailing controls. The size of each dot indicates the percentage of nuclei expressing the respective gene. Adjusted P values from *EdgeR* analysis are provided at the top of each dot. **(H)** Venn diagram illustrating the overlap in DEGs between human tissue and hiPSC derived fibroblasts. Genes tested in both snRNA-seq (N=8863) are shown. **(I)** Dot plot of pro-fibrotic genes within the annotated cell states. Nuclei were grouped by their genotypes and cell states. Gene expression was compared within each cell state. Adjusted P values from *EdgeR* analysis are shown at the top of each dot. CM: Cardiomyocytes. FB: Fibroblasts. EC: Endothelial cells. NC: Neural Cells. AD: Adipocytes. **** P<0.0001, *** P<0.001, ** P<0.01, * P<0.05.

Supplemental Methods

Antibody List	Vendor	Catalog Number
Alpha smooth muscle Actin	Abcam	ab7817
BAG3	Proteintech	10599-1-AP
Beta-Actin	Cell Signaling Technology	5125S
Cardiac troponin (TNNT2)	Abcam	45932
Collagen I	Abcam	ab34710
Connexin 43	Abcam	ab11370
DDR2	Santa Cruz	sc-81707
FLAG	Sigma Aldrich	F1804
GAPDH	Cell Signaling Technology	3683
HSP70	Proteintech	10995-1-AP
HSPB8	Proteintech	15287-1-AP
LC3B-I/II	Abcam	ab48394
p38 MAPK	Cell Signaling Technology	8690
p44/42 MAP kinase (phosphorylated Erk1/2)	Cell Signaling Technology	9101
p44/42 MAPK (Erk1/2) Rabbit mAb	Cell Signaling Technology	4695
phospho-p38 MAPK	Cell Signaling Technology	4511
phospho-SMAD2	Cell Signaling Technology	3108
phospho-SMAD3	Cell Signaling Technology	9520
phospho-TGFBR1	ThermoFisher	PA5-40298
TCF21	Millipore Sigma	HPA013189
TGFBR2	ThermoFisher	701683
Total SMAD2/3	Cell Signaling Technology	3102
Total TGFBR1	Abcam	ab235578
V5-Tag	Cell Signaling Technology	13202
WT-1	Abcam	ab89901
α-actinin (ACTN2)	MACS	130-119-766
Donkey Anti-Rabbit IgG (H+L) Alexa Fluor 594	Invitrogen	A21207
Goat Anti-Mouse IgG (H+L) Alexa Fluor 594	Invitrogen	A11032
Goat Anti-Rabbit IgG (H+L) Alexa Fluor 647	Invitrogen	A21245
Goat Anti-Mouse IgG (H+L) Alexa Fluor 647	Invitrogen	A21235
Anti-Rabbit IgG HRP-linked	Cell Signaling Technologies	7074
Anti-Mouse IgG HRP-linked	Cell Signaling Technologies	7076
Phalloidin Alexa Fluor 488	Invitrogen	O7466

PCR Primers	Sequence (5’- 3’)
ACTA2 Fw	CCGACCGAATGCA GAAGGA
ACTA2 Rev	ACAGAGTATTTGC GCTCCGAA
COL1A1 Fw	GATTCCCTGGACC TAAAGGTGC
COL1A1 Rev	AGCCTCTCCATCT TTGCCAGCA
FN-EDA Fw	TCCAAGCGGAGA GAGT
FN-EDA Rev	GTGGGTGTGACCT GAG
GADPH Fw	GGACTCATGACCA CAGTCCATG
GAPDH Rev	CAGGGATGATGTT CTGGAGAGC
POSTN Fw	TGCCCTGGTTATA TGAGAATGGAAG
POSTN Rev	GATGCCCAGAGT GCCATAAACA

Plasmids list	Vendor	Catalog Number
BAG3-3XFLAG	Vectorbuilder	Vectorbuilder VB210404-1020mex
BAG3[E455K]-3XFLAG	Vectorbuilder	Vectorbuilder VB210404-1020mex
V5-TGFBR2	Vectorbuilder	Vectorbuilder VB210510-1151rca
Empty Vector control	GeneCopoeia	Cat# EX-NEG-M46
siTGFBR2	ThermoFisher Scientific	4390824; AssayID: S14077 silencer select
siBAG3	ThermoFisher Scientific	4390824; AssayID: s18292 silencer select
SBE40-Luc	Addgene	#16495
Silencer Select Negative Control no. 1 siRNA (siCtrl)	ThermoFisher Scientific	4390843
pRL	Promega	E2261
