# Supplementary material for: Cardiac fibroblast BAG3 regulates TGFBR2 signaling and fibrosis in dilated cardiomyopathy
Source: J Clin Invest. 2025 Jan 2;135(1):e181630. doi: 10.1172/JCI181630 (PMC11684812; doi:10.1172/JCI181630)

Supplemental Data 3

Uncropped Western Blots

Ladder Used: PageRuler 26616 unless otherwise specified

Western blots were imaged using Licor Odyssey Fc system at 600 and Chemi channels. The exposures are overlaid in order to show ladder and the outline of the membrane. Due to lane constraints, samples from one experiment may have been run using identical loading volumes across multiple gels and membranes. Some blots are stripped and restained i.e., for total protein after phospho-blot or for GAPDH.

Figure 3A

BAG3

Replicate 1 | Replicate 2 | Replicate 3

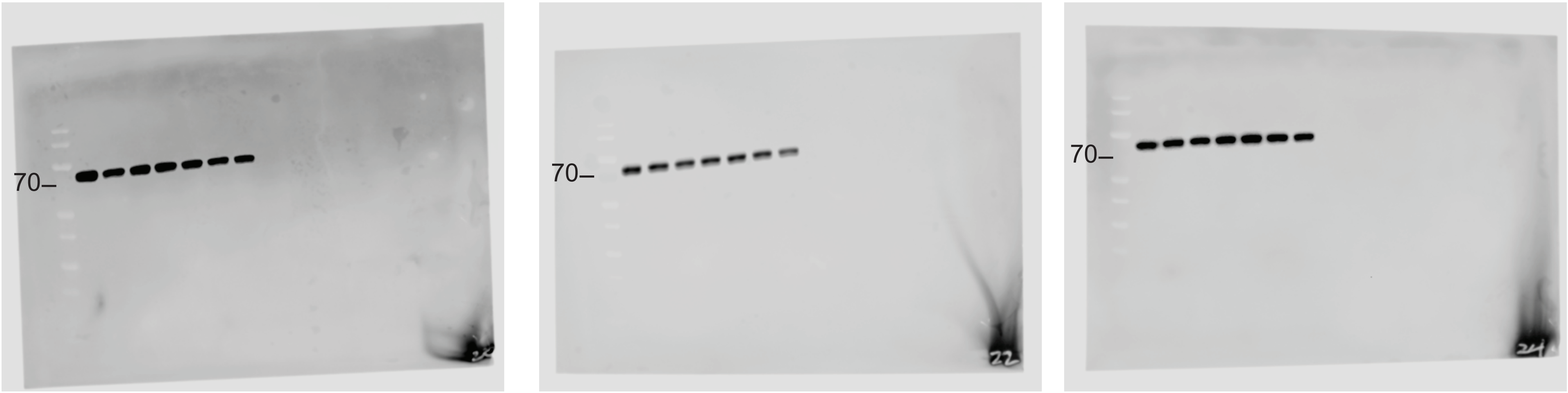

Figure 3A (Cont)

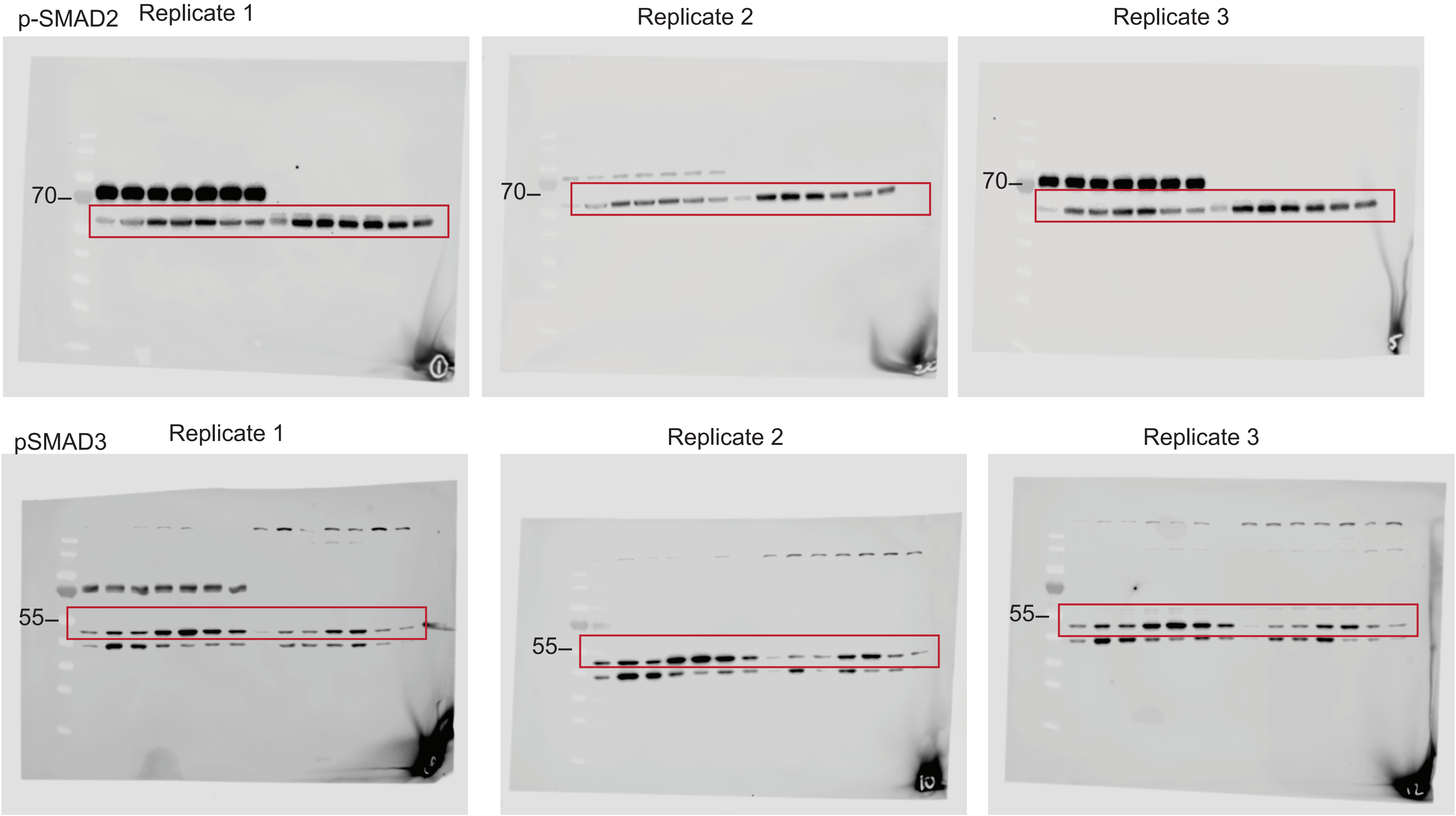

Figure 3A (Cont)

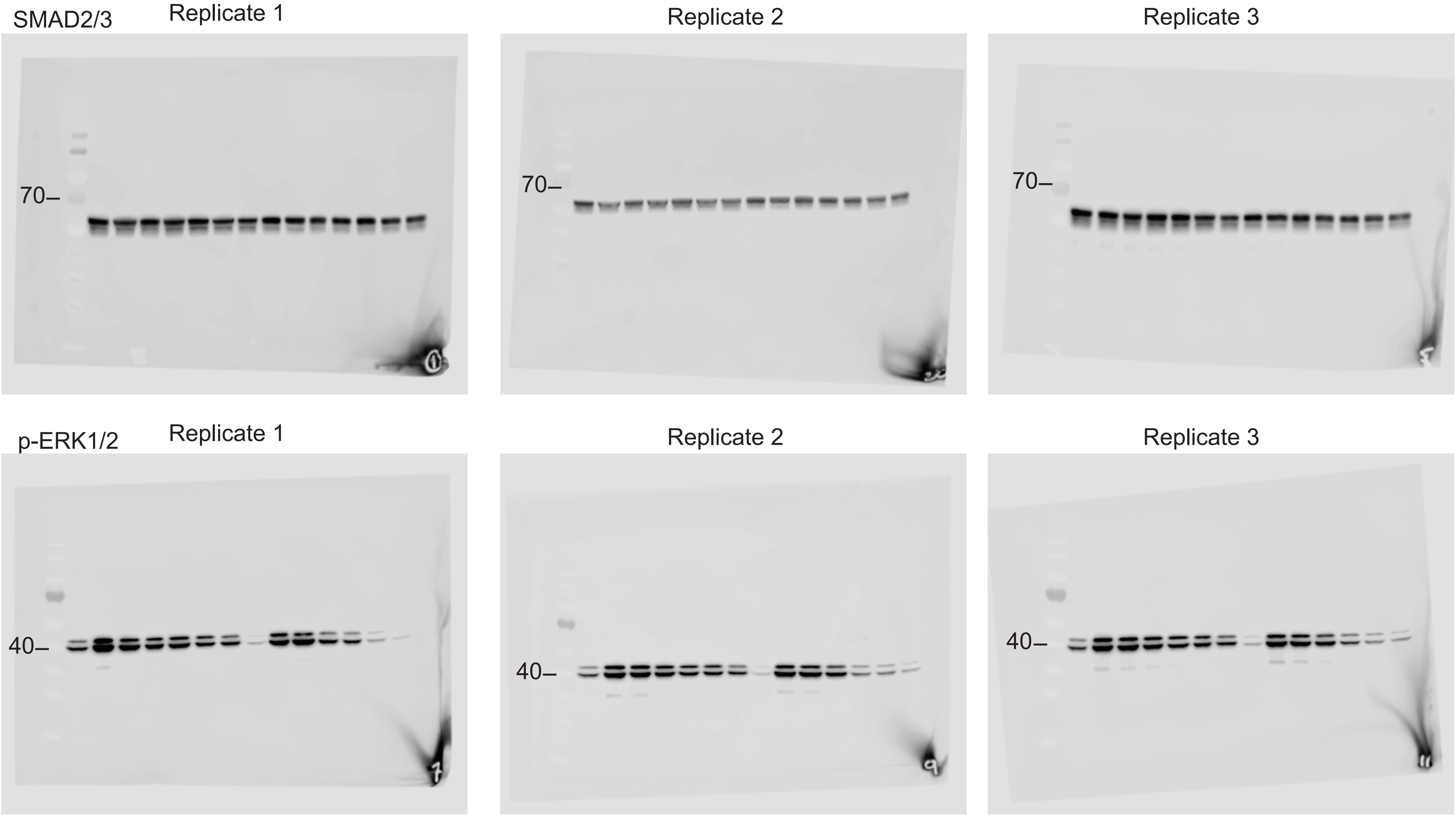

Figure 3A (Cont)

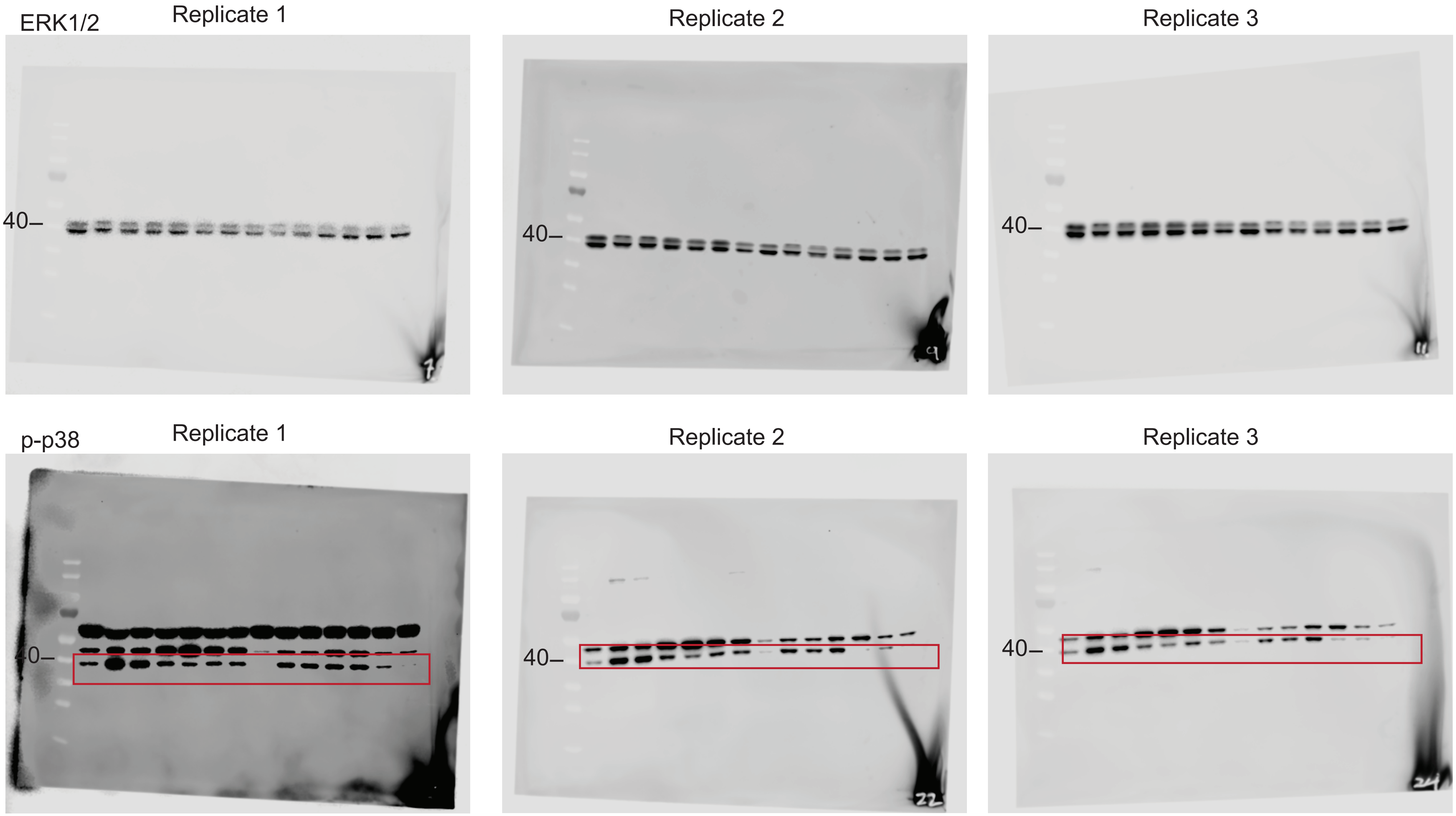

Figure 3A (Cont)

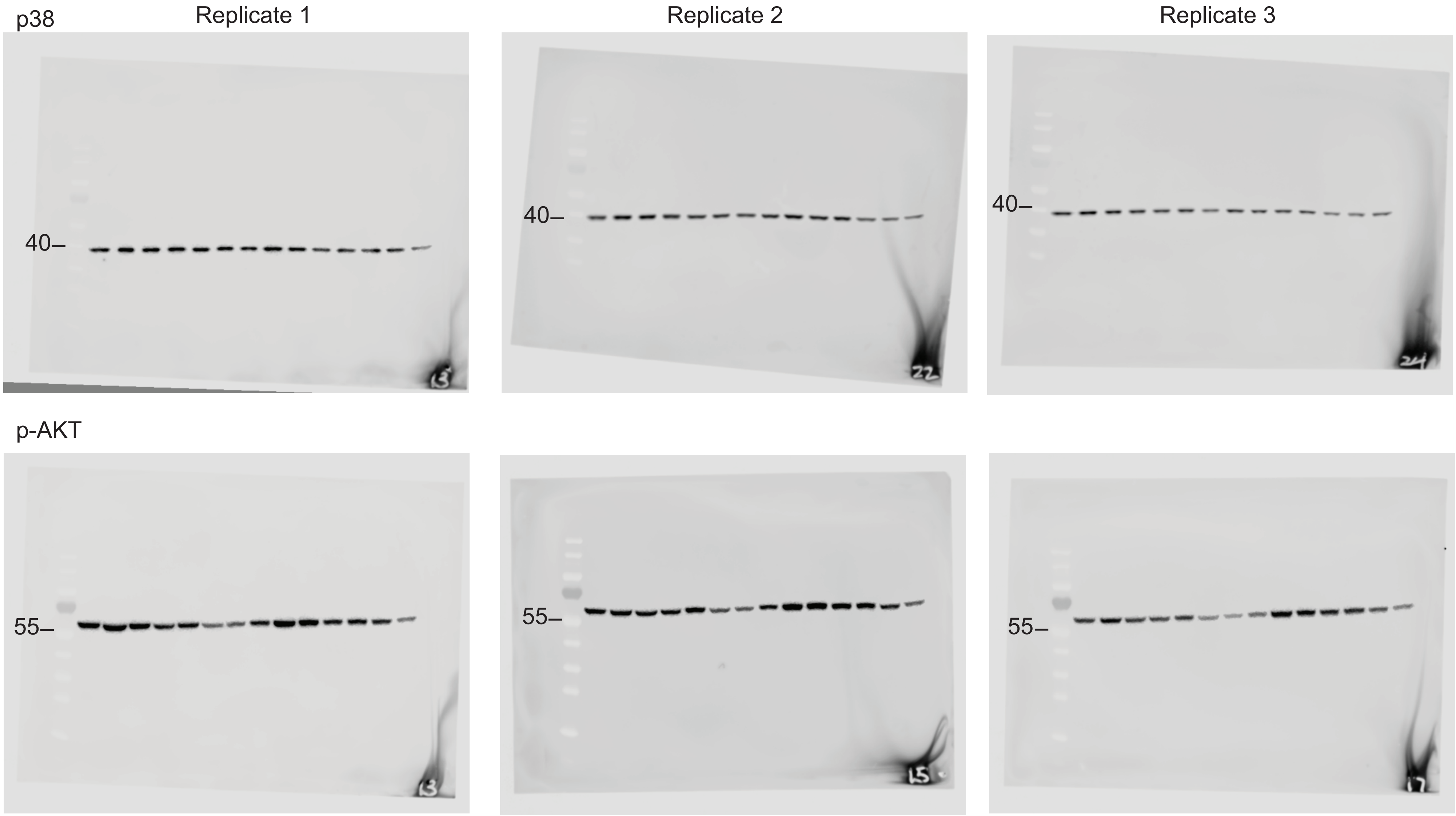

Figure 3A (Cont)

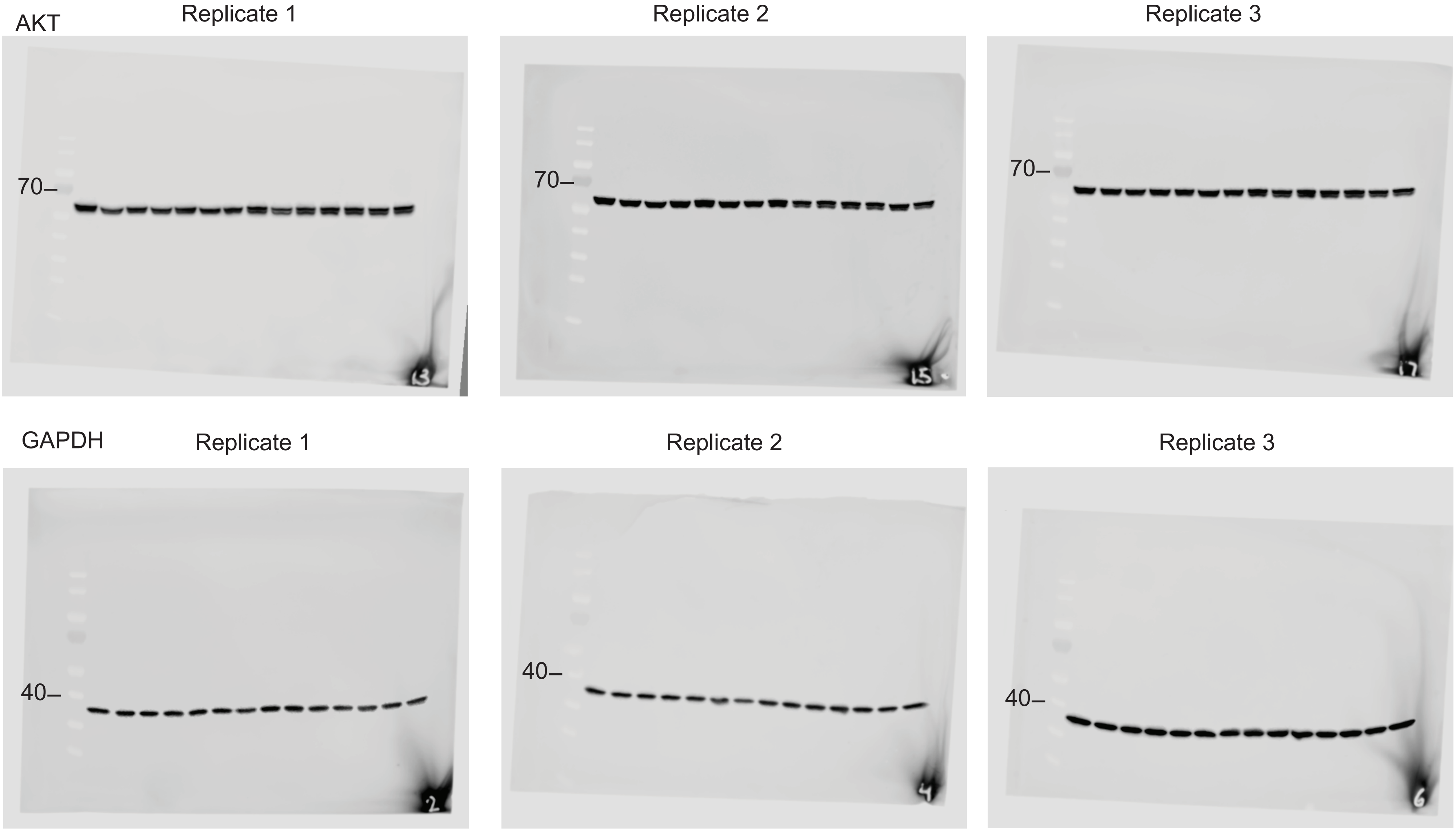

Figure 3I

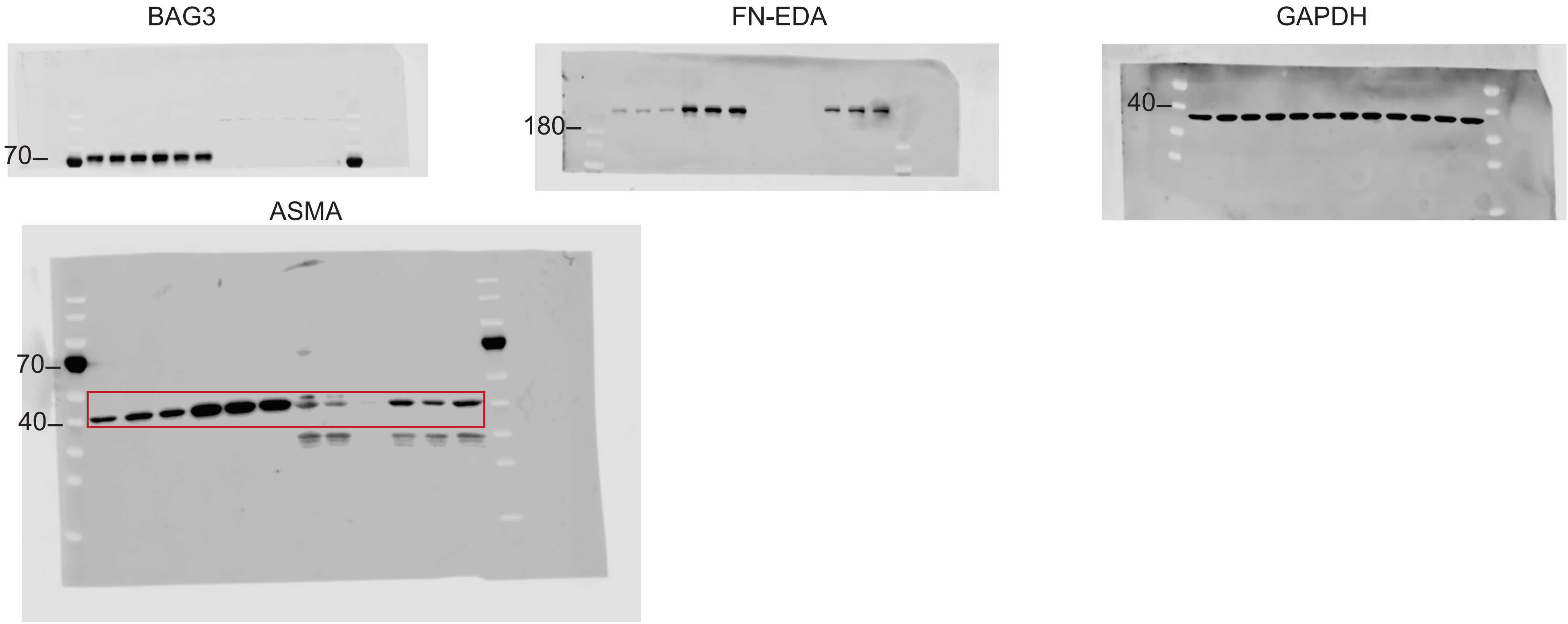

Figure 3J

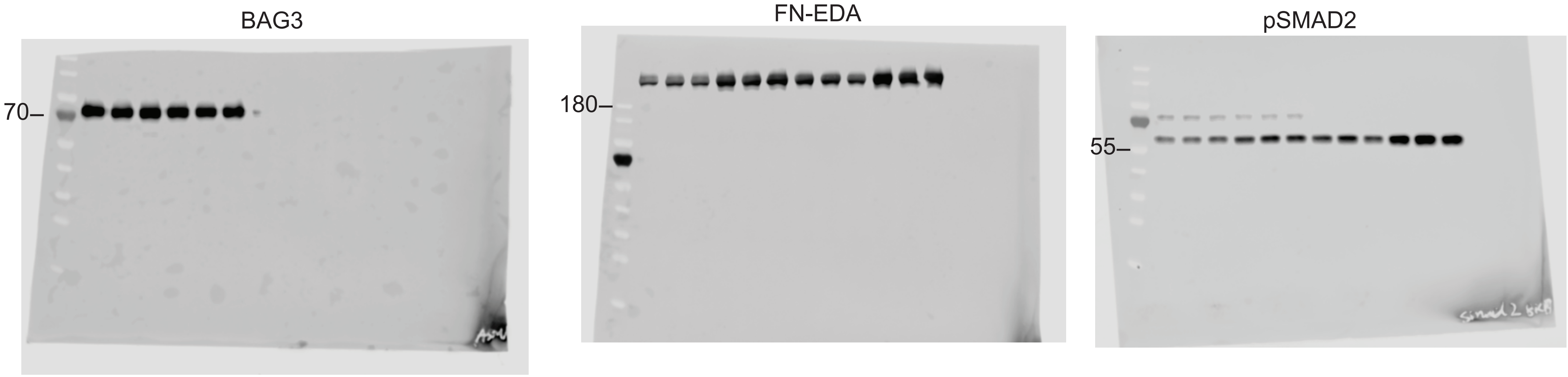

Figure 3J (cont)

ASMA

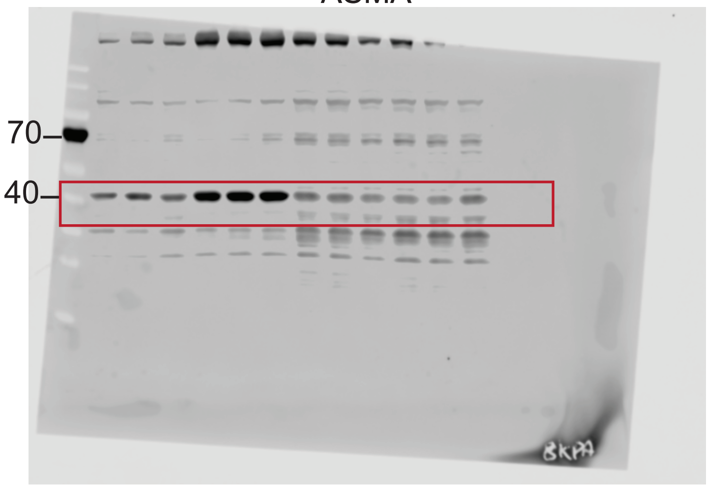

pSMAD3

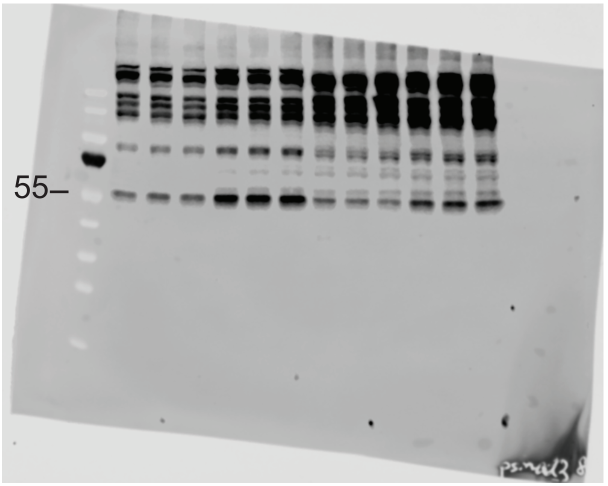

SMAD2/3

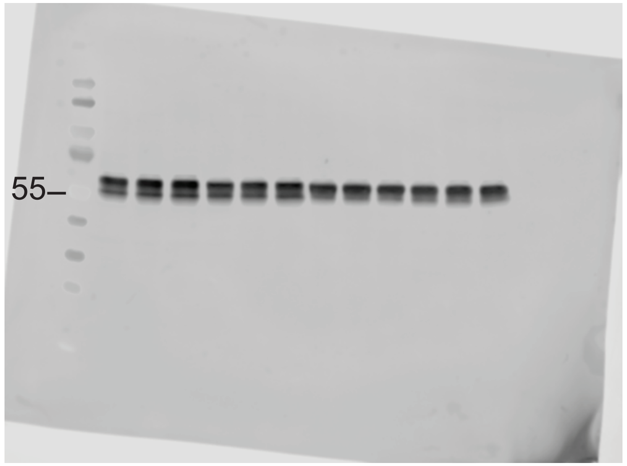

GAPDH

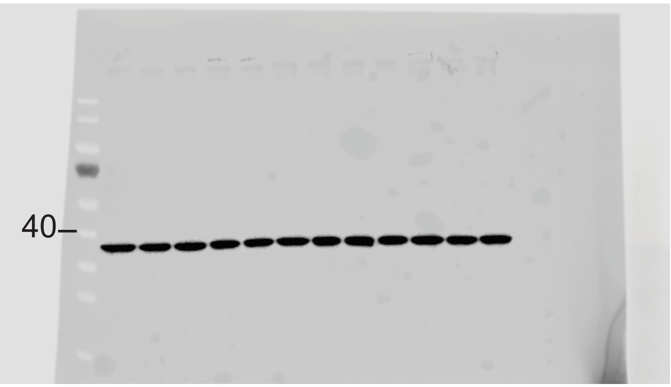

Figure 4B

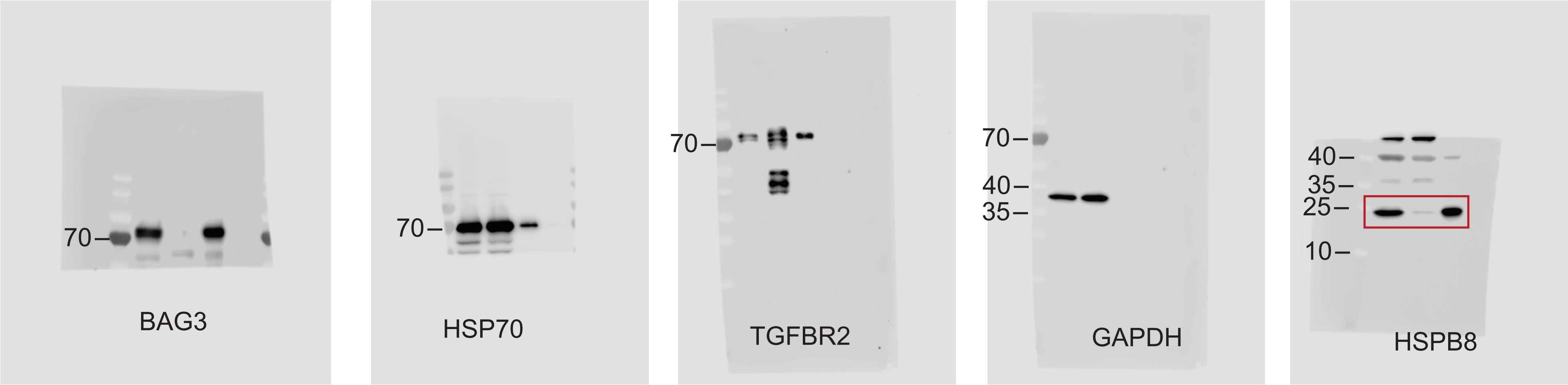

Figure 4C

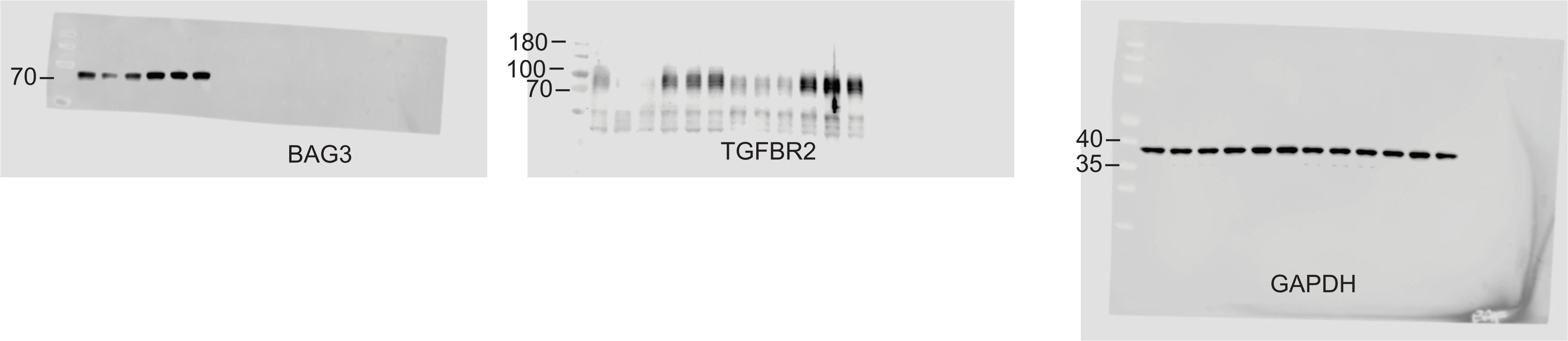

Figure 4D

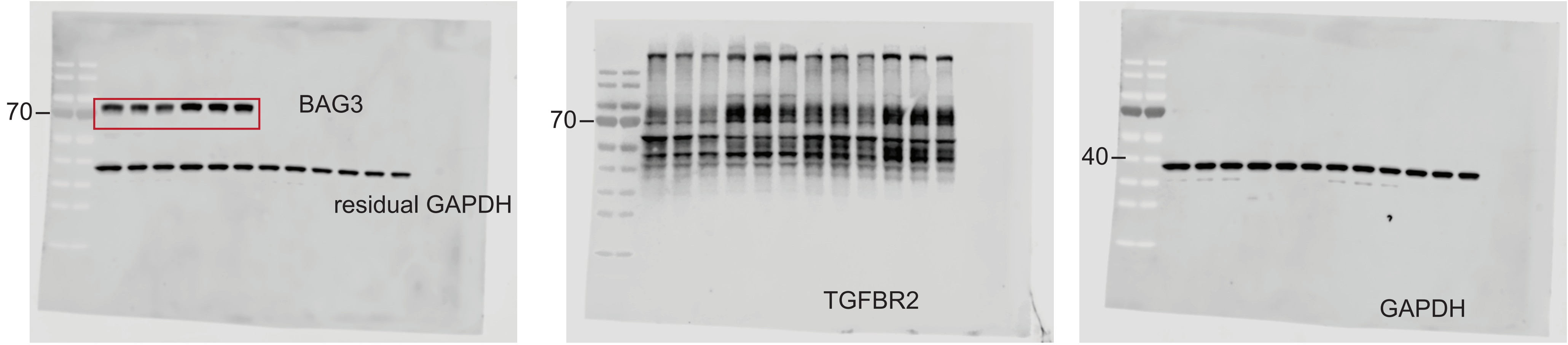

Figure 4G

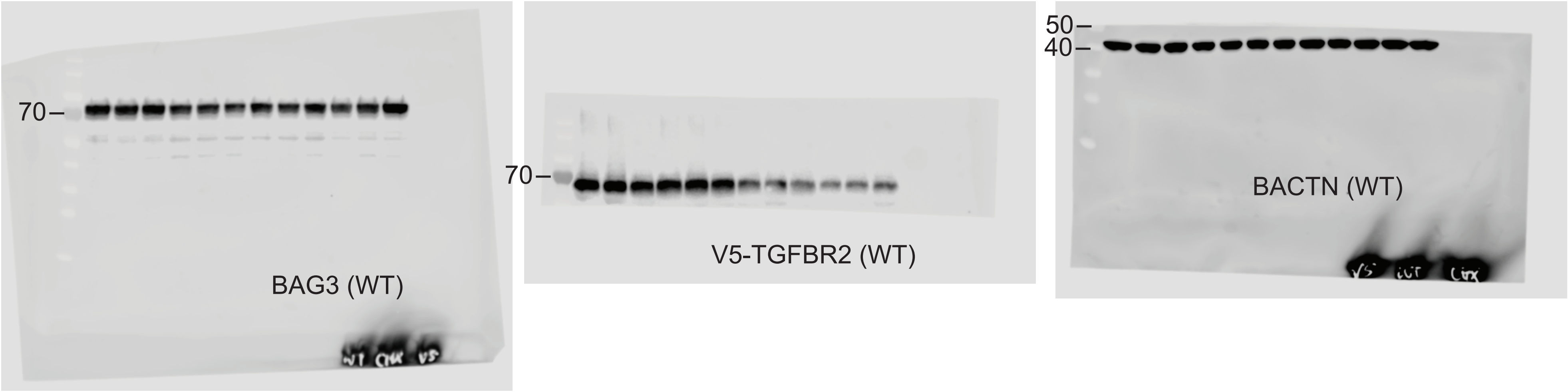

Figure 4G (Cont)

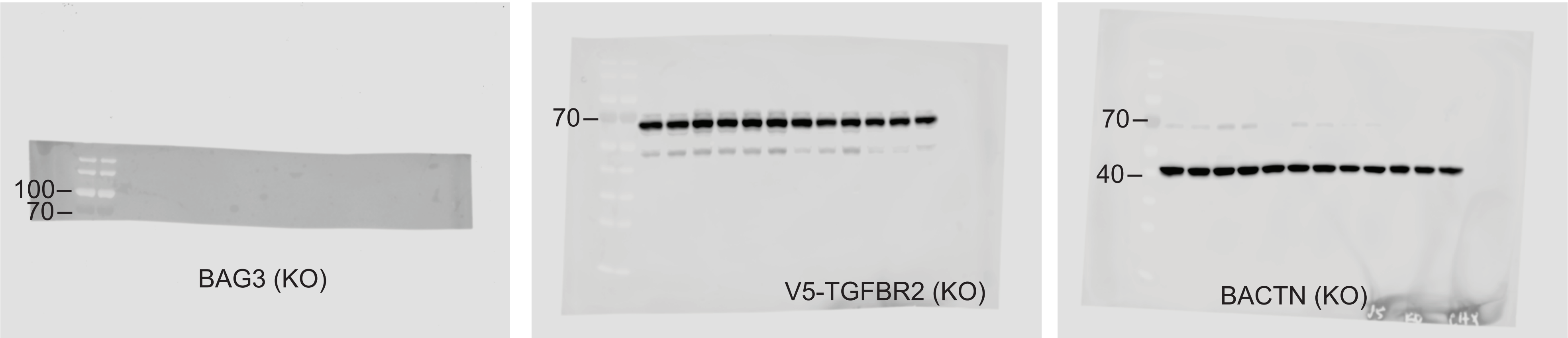

Figure 4I

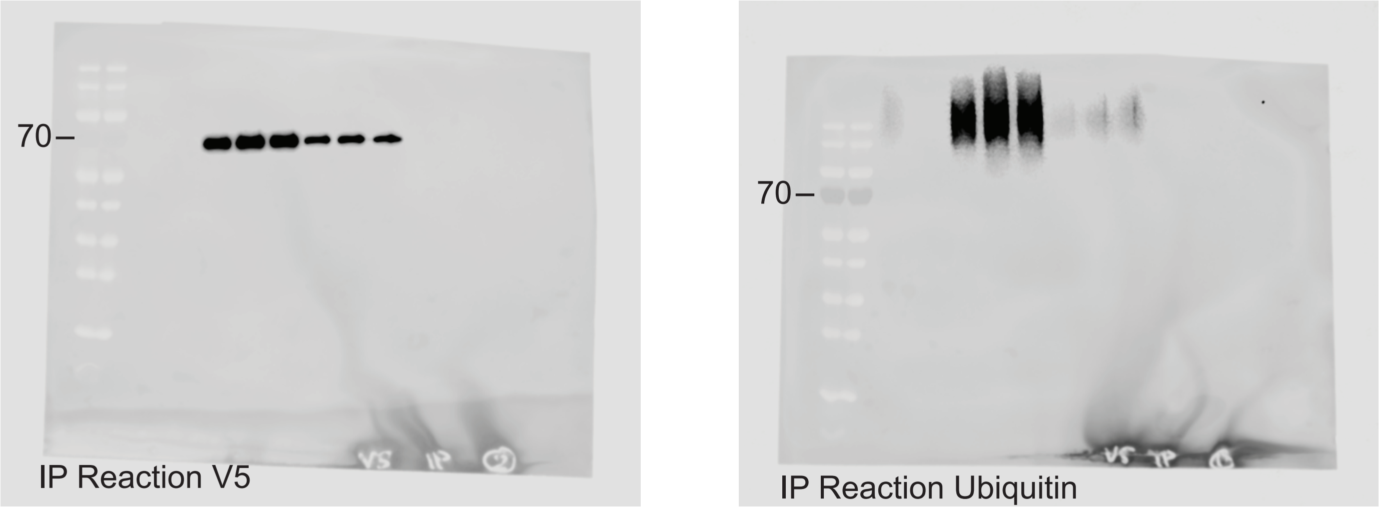

Figure 4I (Cont)

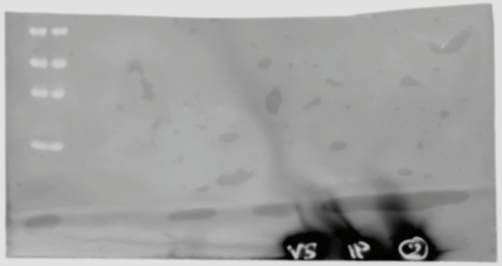

IP Reaction GAPDH

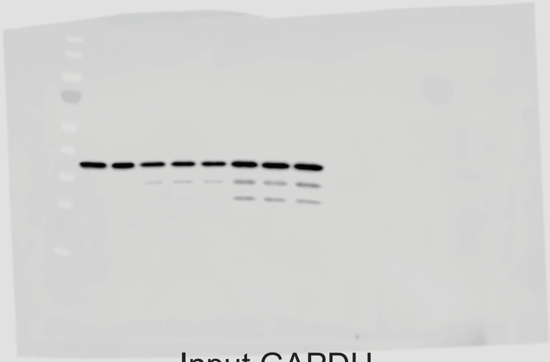

Input GAPDH

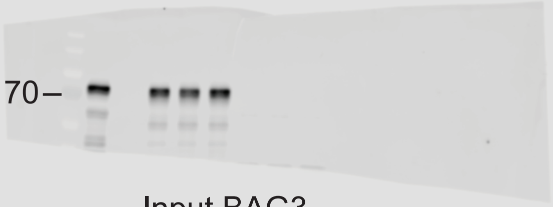

Input BAG3

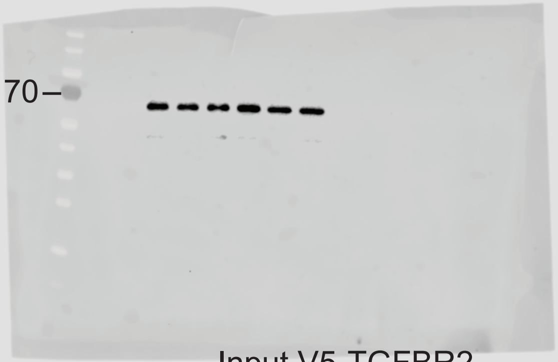

Input V5-TGFBR2

Figure 4K

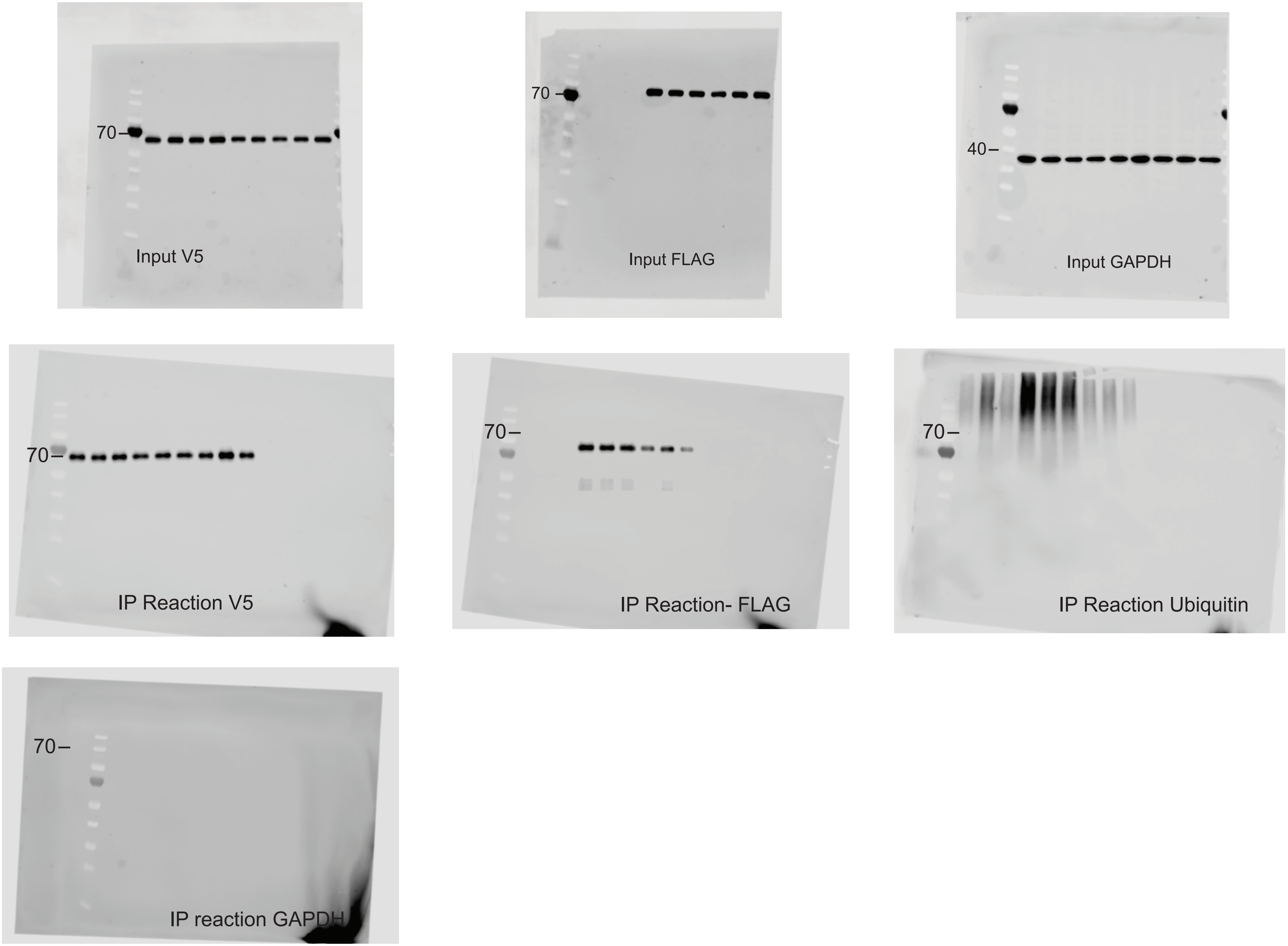

70
Input V5
70
Input FLAG
40
Input GAPDH
70
IP Reaction V5
70
IP Reaction- FLAG
70
IP Reaction Ubiquitin
70
IP reaction GAPDH

Supplemental Figure S1A

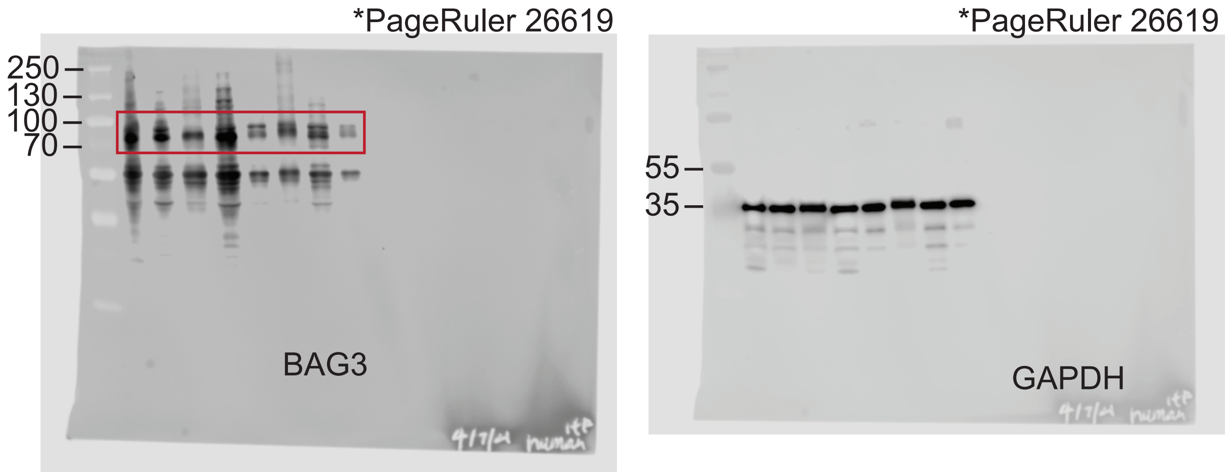

Supplemental Figure S1C

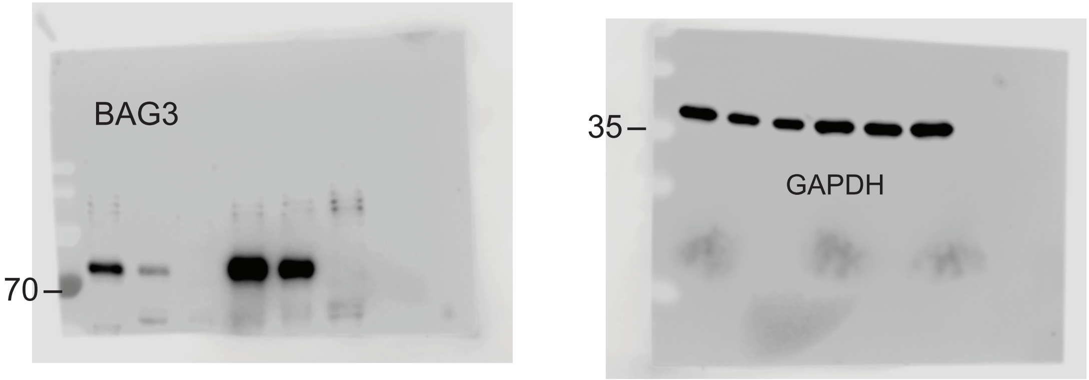

Supplemental Figure S1F

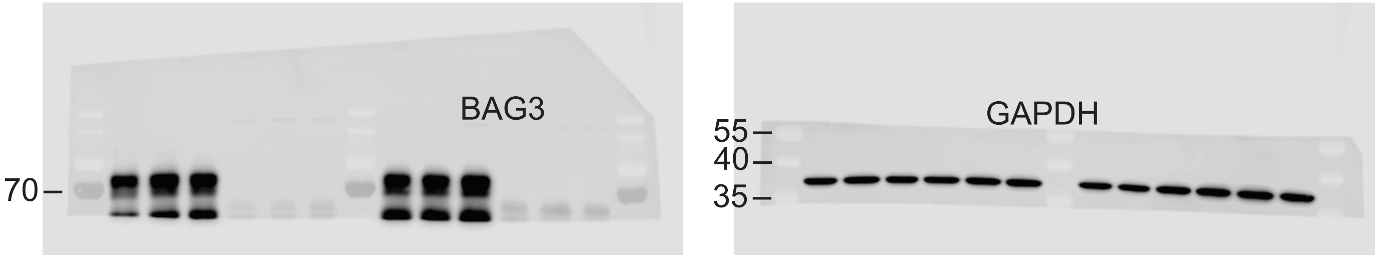

Supplemental Figure 2C

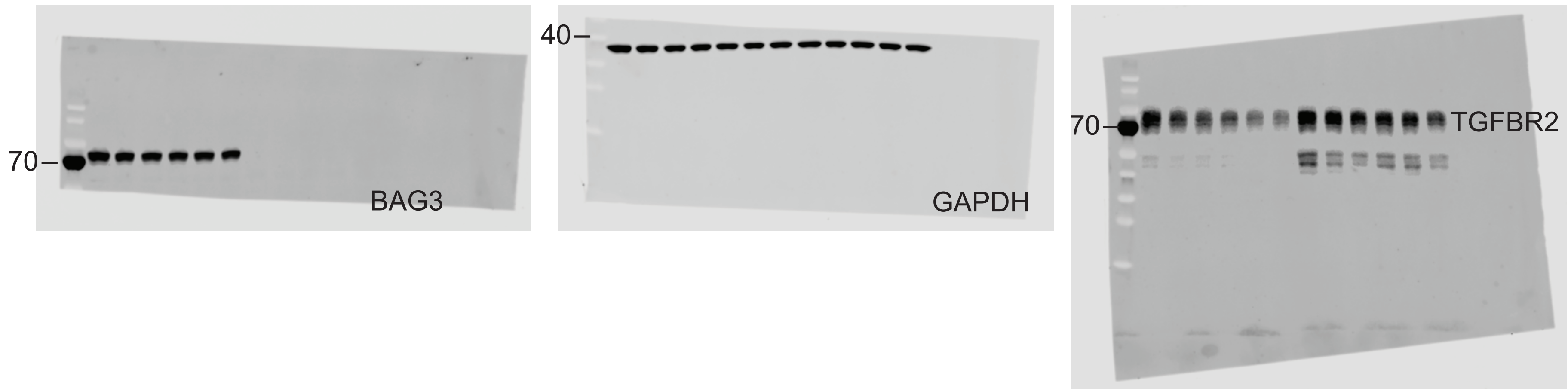

Supplemental Figure S2F

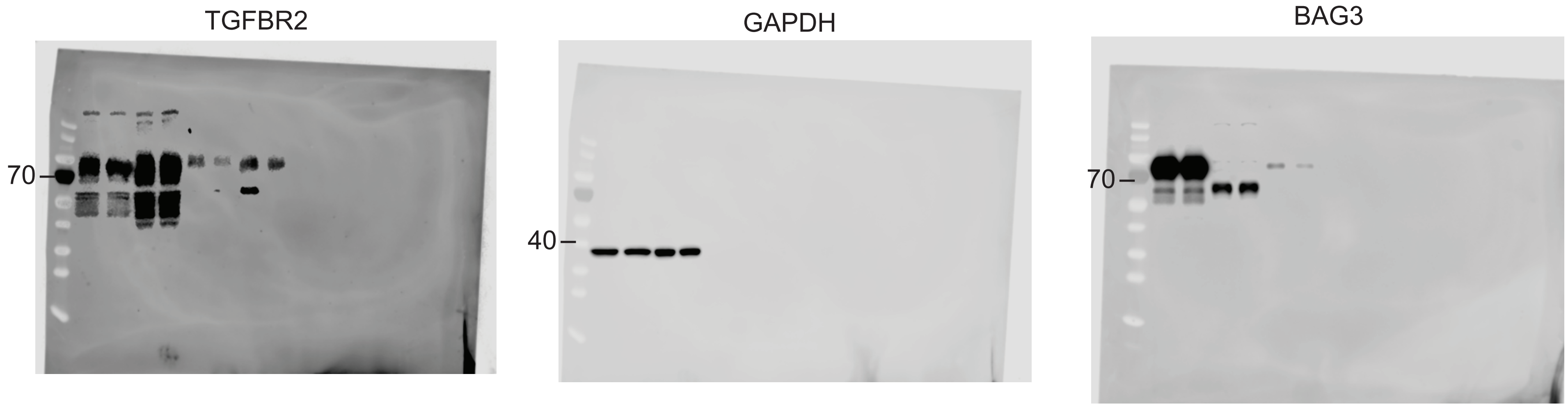

Supplemental Figure S3F

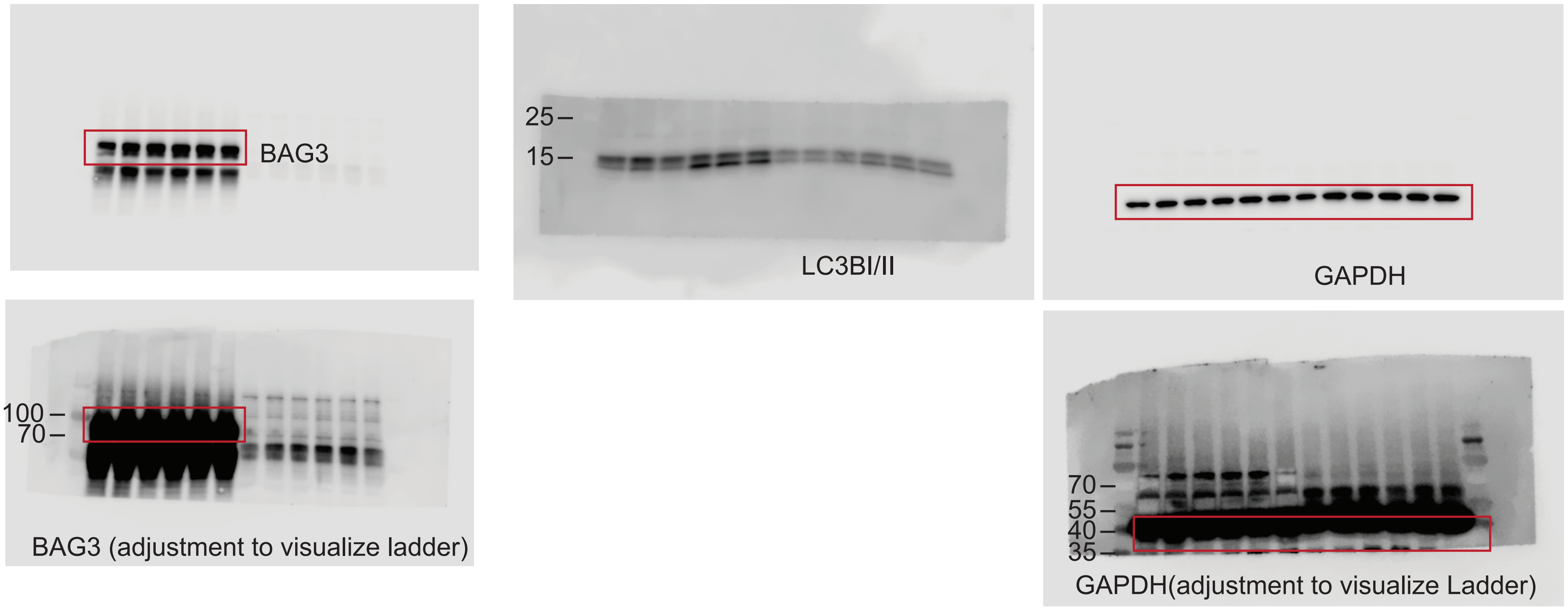

Supplemental Figure S3H

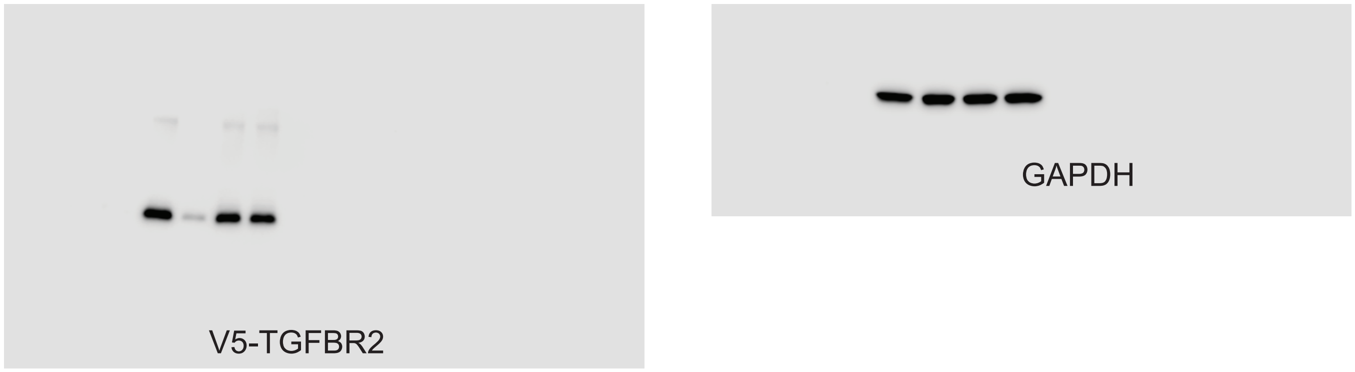

Supplemental Figure S3I

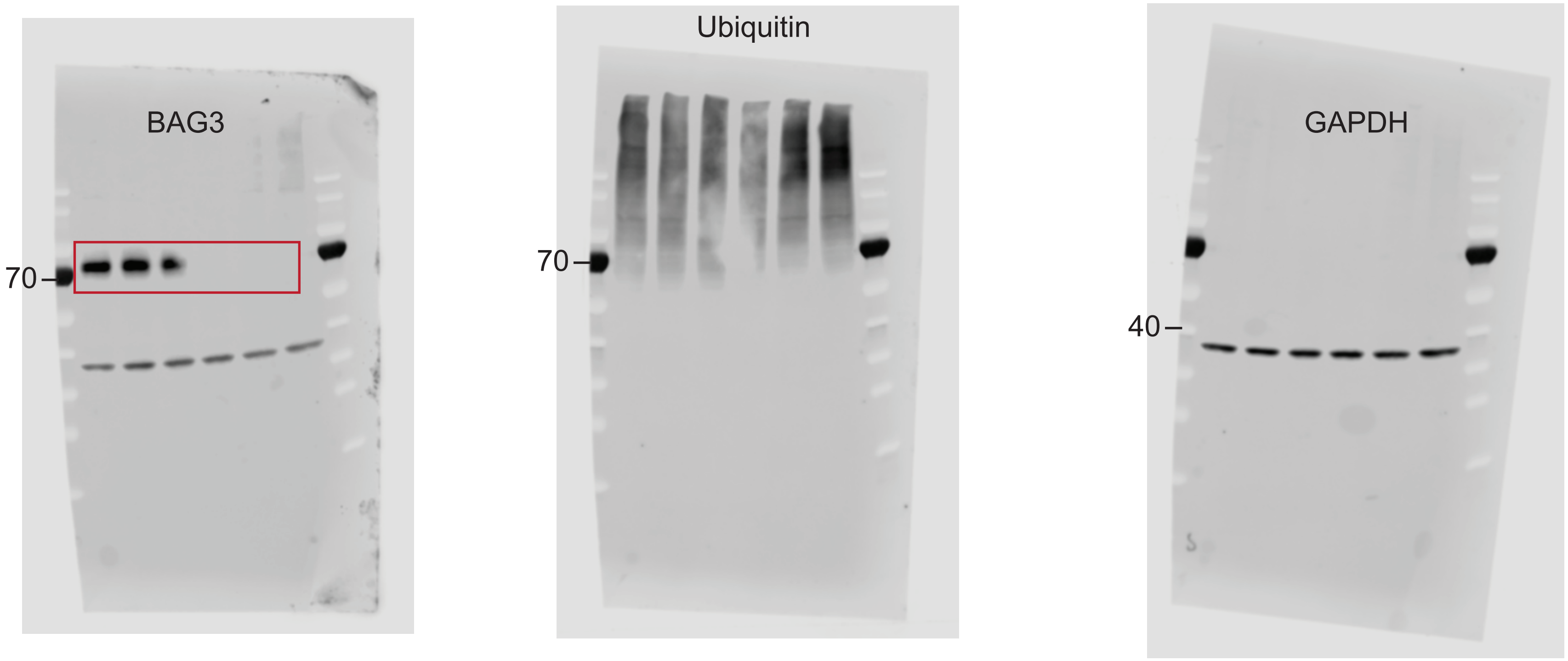

Supplemental Figure S3J

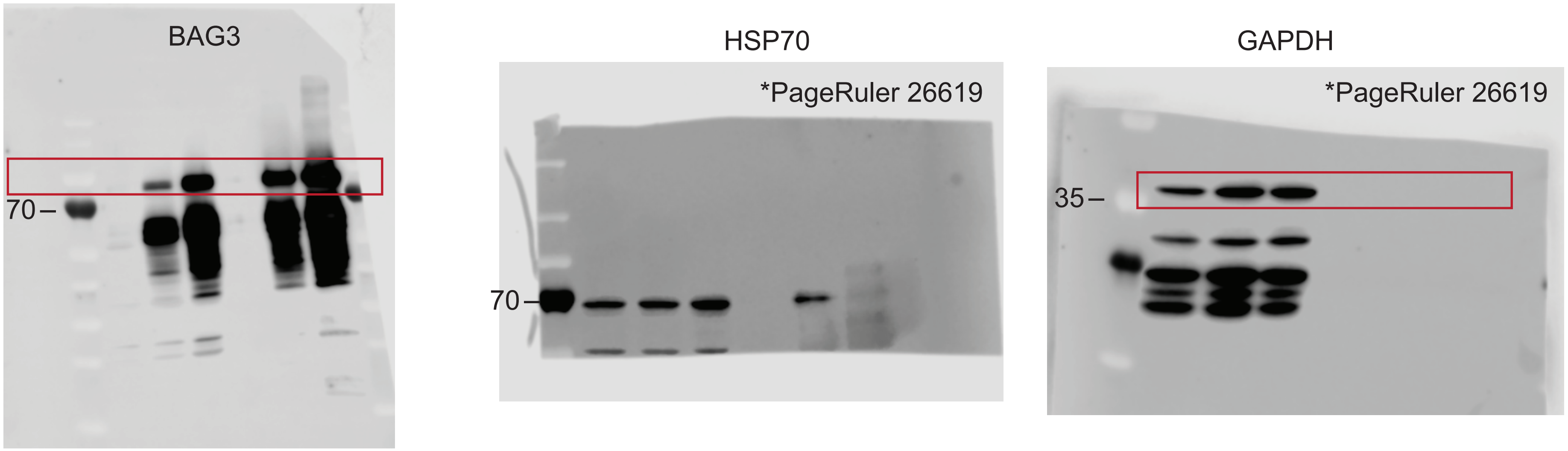

Supplemental Figure S4D

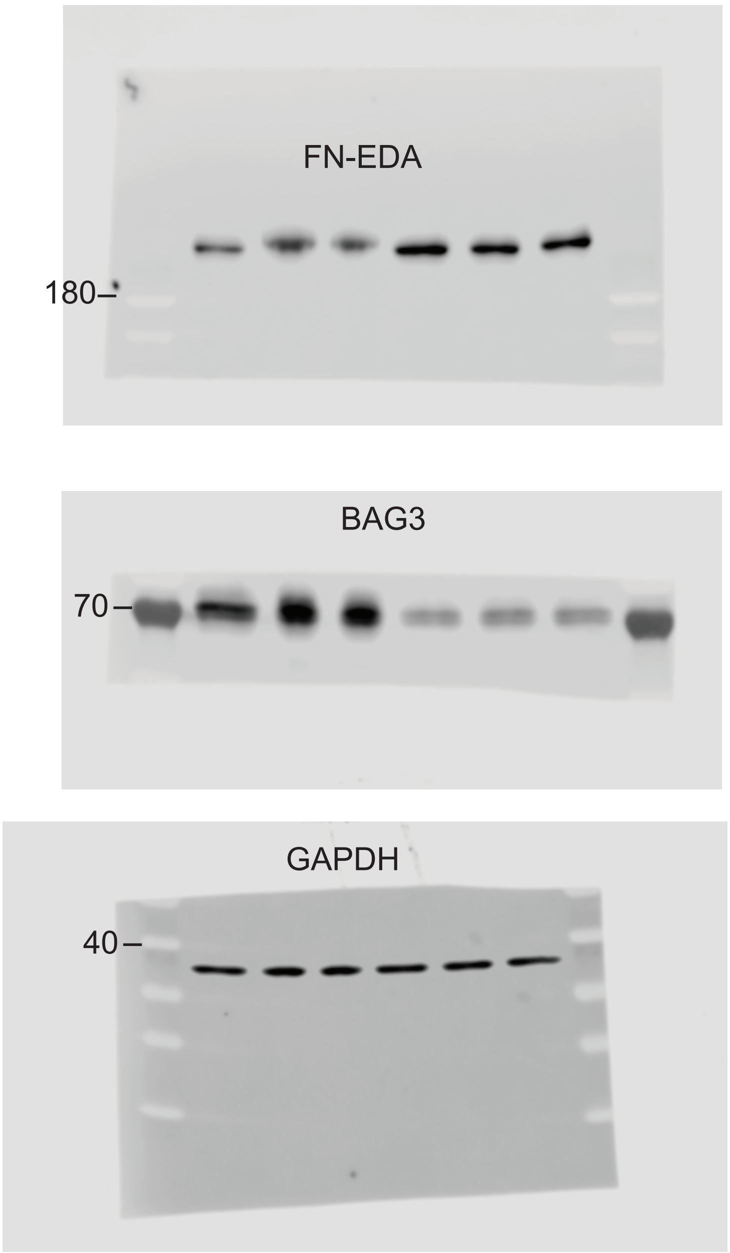

Supplemental Figure S4H

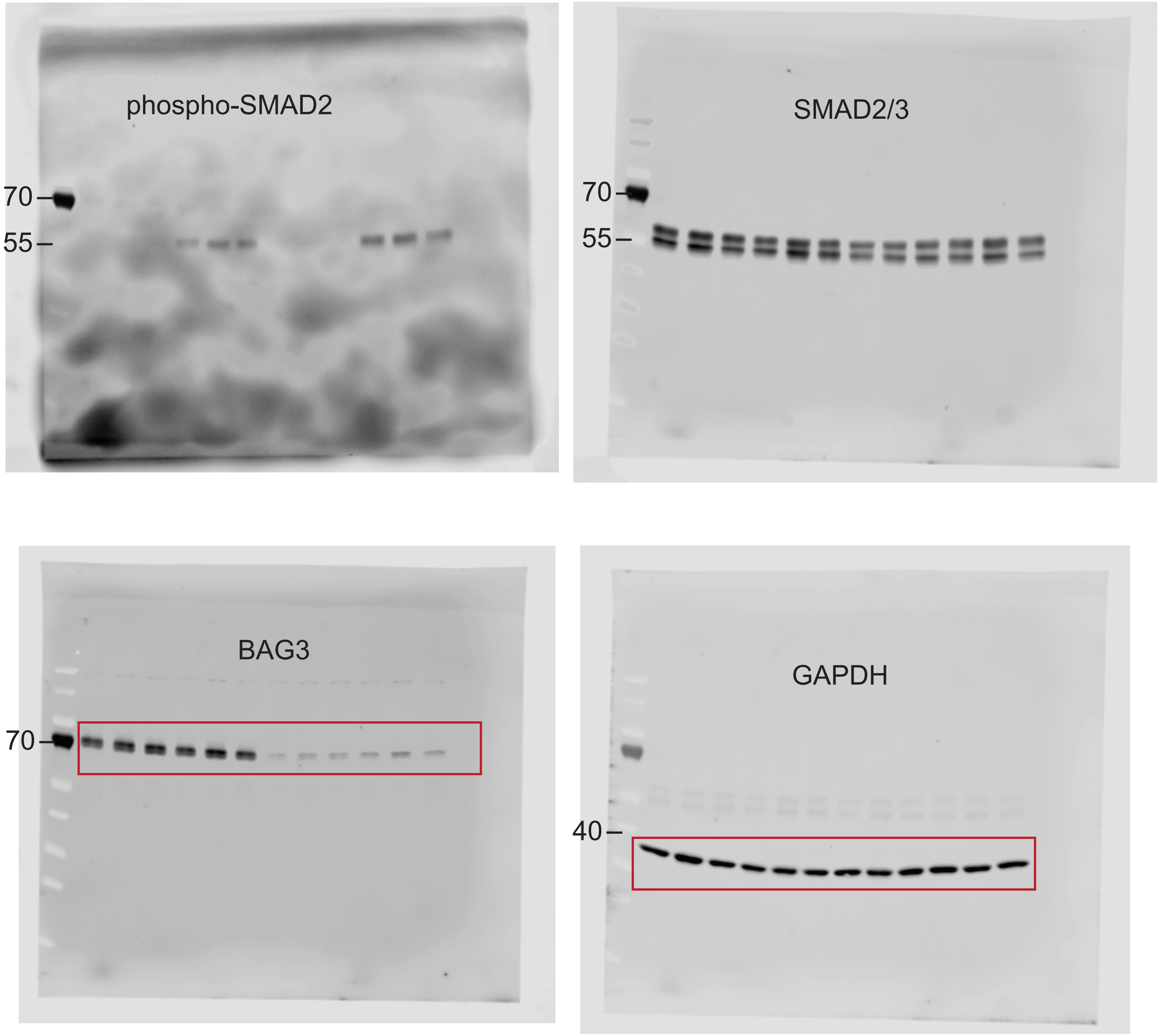

Supplement: Unedited blot and gel images [file jci-135-181630-s033.pdf]
